# Temporal and spatial comparison of food web structure in marine pastures in the Pearl River Estuary: Implications for sustainable fisheries management

**DOI:** 10.1002/ece3.8903

**Published:** 2022-05-13

**Authors:** Peng Xu, Weiguo Zhou, Mujiao Xie, Dewen Ding, Anning Suo

**Affiliations:** ^1^ CAS Key Laboratory of Tropical Marine Bio‐Resources and Ecology South China Sea Institute of Oceanology Chinese Academy of Sciences Guangzhou China; ^2^ University of Chinese Academy of Sciences Beijing China; ^3^ Southern Marine Science and Engineering Guangdong Laboratory (Guangzhou) Guangzhou China

**Keywords:** artificial reefs, ecosystems, fishing moratorium, food webs, keystone groups

## Abstract

The biological and ecological integrity of marine ecosystems in the Pearl River Estuary (PRE) has been compromised due to overfishing and water pollution. Fishing moratorium and artificial reef construction have been implemented in Wanshan and Miaowan for resource protection and restoration. Therefore, food web structure and trophic pathways of Wanshan, Miaowan, and Wailingding in different temporal and spatial situation will be determined using the Ecopath model, as well as the keystone species affecting these ecosystems, which can provide a basis for fishery management. The results showed that the energy transfer efficiency of IV and V trophic levels (TL) was higher than that of II and III‐TL before and after fishing moratorium, and the energy transfer efficiency of artificial reefs II and III‐TL was only slightly higher than that of nonartificial reefs in Wanshan. In addition, the mean values of ecosystem property indicators (consumption, respiration flow, total system throughput, and total biomass) after the fishing moratorium were significantly higher than those before the fishing moratorium. The average value of the ecosystem attribute indicators (consumption, respiration flow, total system throughput, and total biomass) of artificial reefs is lower than those of nonartificial reef areas, which may be related to the differences in community composition between artificial reefs and non‐artificial reefs. Finally, *Nemipterus japonicus* and *Gastrophysus spadiceus* are keystone species that distinguish the Wanshan and Miaowan artificial reefs from other areas. Overall, the fishing moratorium has a positive effect on the short‐term restoration of fishery resources, mainly restoring short‐life cycle organisms. However, the construction of artificial reefs will be more conducive to the persistence of ecosystem restoration. In addition, reasonable proliferation, release and fishing of *N. japonicus* and *G. spadiceus* will be beneficial to the sustainable utilization of fishery resources.

## Graphical abstract

This article evaluates the nutritional status and development stages of seven different spatio‐temporal models. The modeling conclusions are biologically reasonable, and can provide an effective tool for analyzing the structure and evolution of ecosystems and testing the nutritional effects of different species, and provide a basis for further research on management in the future.

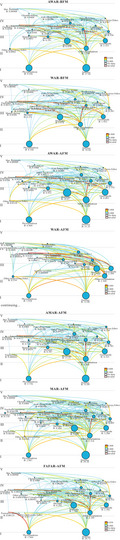

## 1 INTRODUCTION

In the past, fishery management had been based on the dynamic investigation of individual populations (Hilborn & Walters, 2013). The past few decades, however, have seen a growing body of evidence that trade‐offs must be considered when multiple species are exploited simultaneously (Christensen & Walters, 2004), especially the trophic structure and flows of biomass through species interactions (Christensen & Pauly, 1995). Furthermore, predator–prey relationships are used to capture the interdependencies between multiple exploited populations. However, only focusing on commercial species, they were unable to address the dynamics of low‐trophic organisms (phytoplankton, zooplankton, and benthos) affecting commercial species. These aspects need to be considered in an in‐depth discussion of the structure and function of the marine food web in order to develop rational management options (Staebler et al., 2018). Measurements of biomass transfer and trophic efficiency between functional groups (FGs) provide needed information for evaluating the impact of changes on some groups and their interrelations within the ecosystem via trophic interactions (Christensen & Pauly, 1993; Christian et al., 1996; Ulanowicz, 2012). The Ecopath model (Ecopath with Ecosim [EWE]) has been proven capable of capturing real ecosystem dynamics in a variety of ecosystems (Brown et al., 2010; Walters et al., 2005). Campbell et al. also developed and applied a multispecies, individual‐based model to study the long‐term effects of increasing numbers of artificial reefs (ARs) on fish abundance and biomass. They found that increased numbers of ARs generally produced higher biomass, but at the cost of slower growth and smaller individual (Campbell et al., 2011).

The Pearl River Estuary (PRE) has experienced overfishing and water pollution over the past two decades, and the deteriorating environmental conditions have affected the biological and ecological integrity of the estuarine ecosystems (Huang et al., 1997; Ke et al., 2007; Li & Huang, 2008). To restore the declining fishery resource and the damaged ecosystem, the Chinese government has adopted a series of policies and engineering technologies, including a fishing license system, a summer moratorium, fierce restrictions on illegal fishing, a construction of marine ranching, and other measures (Yu & Yu, 2008). The release of ARs is one of the important manifestations of the construction of marine ranching. AR are broadly defined as any underwater structures placed on the bottom layer to mimic certain characteristics of natural reefs, altering the physical, biological and/or socioeconomic variables associated with marine resources (Seaman & Jensen, 2000). A large number of studies have shown that the introduction of fishing moratoriums and ARs can gather organisms in the reef area, which can conserve and enhance fishery resources (Fabi & Sala, 2002; Wang et al., 2018). The earliest documented AR occurred in the 1830s, when logs from huts were used to the coast of South Carolina, United States, to improve fishing yield (Weisburd, 1986). AR is a widely used tool for fisheries restoration and has been researched extensively. For example, Pickering et al. discussed the potential of AR as a tool to aid in the restoration of coastal ecosystems (Pickering et al., 1998). Cresson et al. analyzed the gastric contents of 23 fish species collected on ARs to assess their trophic niche and feeding behavior. Their results showed that neither the diet nor the nutrient network structure of fish was altered on ARs compared to the natural environment (Cresson et al., 2014). Lv et al. studied the distribution of macrobenthic biodiversity on oyster reefs in the Yangtze Estuary. The result shows that water salinity and substrate factors are the main indicators affecting the distribution of macrobenthos (Lv et al., 2016). However, there are still many unknown areas in the study of ARs, including that researchers are still unaware of using ecological theory to explore the impact of ARs on ecosystems (Bortone, 1998). How AR construction can be applied to ecosystem management also needs to be further clarified in further research (Bortone, 1998). In addition, there are relatively few detections of population dynamics in long‐term sequences after the AR are released, especially for quantification of ‘keystone’ species for conservation destinations (Jordan, 2009; Lee & Zhang, 2018).

Based on the above problems, this paper focuses on three main issues for the PRE marine pasture ecosystem: (1) to compare the food webs structure and function of different model; (2) to determine the key trophic pathways and keystone species in the system; and (3) by comparing with previous studies on similar coastal and marine ecosystems, we can understand the characteristics and development status of these ecosystems. This article aims to help fishery managers understand whether the ecosystem functions of artificial reef areas and nonartificial reef areas will continue to change over time and space. In order to solve the above problems, this paper proposes and constructs a steady‐state trophic model with different time and space constraints in the PRE marine ranching area.

## 2 MATERIALS AND METHODS

### 2.1 Study area

**FIGURE 1:**
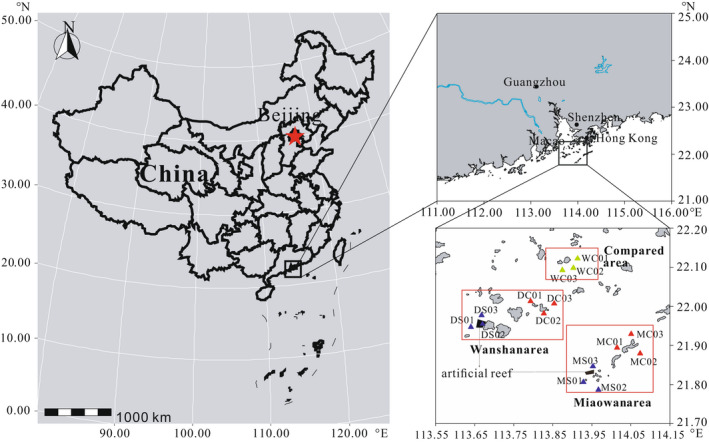
Locations of study sites of the Pearl River Delta coastal area

The PRE coastal ecosystem used in this study, which extends from 111°00′E to 116°00′E, 21°00′N to 22°00′N, has an area of about 43,000 km^2^ (Figure 1). The annual averaged river discharge is 10,524 m^−3^ s^−1^, with 2105 m^−3^ s^−1^ runoff during the dry season from October to March of next year, and with 8419 m^−3^ s^−1^ runoff during rainy season in April to September (Yin et al., 2004; Zhao, 1990). The PRE is an important nursery for many fish species (Qiu et al., 2008), and maintains the important commercial fishery. The Chinese government has built Wanshan marine ranching to restore fishery resources in the PRE.

The study areas of Wanshan, Miaowan, and Wailingding are located in 113°36′–113°52′E, 21°55′–22°03′N, 113°53′–114°06′E, 21°47′–21°57′N, 113°49′–113°58′E, 22°05′–22°09′N, respectively (Figure 1). The construction area of Wanshan and Miaowan AR are 2.5 and 1.63 km^2^, and the average water depth is 20 and 40 m, respectively. The control area was selected as the part of Wailingding without AR.

Studies have shown that the community structure of phytoplankton is different under the influence of the PRE diluted water, which then affects the community structure of fishery resources (Su et al., 2020). Wanshan and Wailingding are located inside PRE due to the influence of fresh water on land, with an average salinity of 32.37 ± 0.02 and 32.52 ± 0.02 (internal data), and Miaowan Island is located at the outer edge of PRE with an average salinity of 33.93 ± 0.01 (Su et al., 2020). Artificial reef construction changes the fishing intensity of the area and provides a good habitat environment for fishery resources (Xiao et al., 2020). Additionally, different reef sizes may also cause differences in regional ecosystems. Therefore, random sampling of stations in the above areas was carried out, 15 sampling sites were set up in Wanshan marine ranching, of which six sites (DS01, DS02, DS03, DC01, DC02, DC03) were deployed in the Wanshan AR area and adjacent area, while six more sites (MS01, MS02, MS03, MC01, MC02, MC03) were deployed in the Miaowan AR area and adjacent area, and three sites (WC01, WC02, WC03) were deployed far away from the AR area (Figure 1, Table 1). This study attempts to explore the structure and function of the food web in the AR and nonartificial reef area of PRE marine ranching.

**TABLE 1:** Comparison of abbreviations and full names of seven different regional models in PRE marine ranching in 2020

Abbreviation	Full name
AWAR‐BFM	adjacent to Wanshan artificial reef area before the fishing moratorium
WAR‐BFM	Wanshan artificial reef area before the fishing moratorium
AWAR‐AFM	adjacent to Wanshan artificial reef area after the fishing moratorium
WAR‐AFM	Wanshan artificial reef area after the fishing moratorium
AMAR‐AFM	adjacent to Miaowan artificial reef area after the fishing moratorium
MAR‐AFM	Miaowan artificial reef area after the fishing moratorium
FAFAR‐AFM	far away from artificial reef area after the fishing moratorium

### 2.2 Principle of model construction

The Ecopath model defines that an ecosystem is composed of a series of ecologically related FGs, and all FGs can cover the process of energy flow in the ecosystem (Pauly & Christensen, 1993). Functional groups can be species with the same ecological habits, important species, or different body lengths or age groups of important species, organic debris, phytoplankton, zooplankton, and benthic organisms. According to the principle of nutrition kinetics, the energy input and output of each FG are balanced (Lin et al., 2009). Ecopath creates a static mass‐balanced snapshot of the resources in a given ecosystem and their interactions. The core component of Ecopath consists of two master equations: one to describe the mass balance and one for the energy balance of each group. Further details on the Ecopath modeling approach can be found in review literature (Christensen & Walters, 2004; Heymans et al., 2016).

### 2.3 Model structure: definition of functional groups

The groups of a system may be an ecologically or taxonomically related species, single species, or size/age groups, which correspond to what are known as ‘‘functional groups’’ (Christensen et al., 2005). In the trophic model of PRE marine ranching, the commercially important fish species were split from omnivorous fish and planktivorous fish, such as flatfishes, *Saurida tumbil*, *Trachurus japonicus*, *Argyrosomus argentatus*, *Psenopsis anomala*, *Thryssa kammalensis*, other Cynoglossidae, other *Trichiurus*, other Synodidae, Tetraodontidae, other Sciaenidae, Mugilidae, Clupeidae, Engraulidae, Anguilliformes, other Piscivorous fish, and other demersal fish. Other uncommercially fish groups were also considered, including Anglerfish, other gobiidae, *Secutor ruconius*, Scorpaenidae, other omnivorous fishes, other benthic fishes 1, and other benthic fishes 2. We also considered the ecologically important species such as marine mammals, Chondrichthyes, and fish‐eating birds as the individual groups as well. These species serve as conservation the targets and nonfish groups in the model. In addition, lower trophic units such as phytoplankton, zooplankton, benthos (including Mantis shrimp, Shrimps, Crabs, Mollusks, and Gastropoda) and detritus are included in the model. Gastropoda and Cephalopods were selected as separate FGs from mollusks. Therefore, our classification resulted in a total of 36 FGs, including 24 fish groups, 6 invertebrate groups, 3 groups of ecological conservation, 3 groups of the primary producers and the secondary consumers. All the FGs were selected to represent the food web structure as shown in Tables S1 and S5.

### 2.4 Data sources

A majority of the input data used in the model were collected from in situ surveys and peer‐reviewed publications. A detailed description of data sources is given in Tables S2 and S3. Biomass data were obtained from different sources: for the fish groups, biomass were estimated by the swept‐area method from bottom‐trawl surveys in April and December 2020 in the PRE by ecosystem ecology research group, South China Sea Institute of Oceanology, Chinese Academy of Sciences (Chen et al., 2015). The biomass of nekton was estimated by the swept area method (Bailey, 1986), and the vertical trawl method was used for phytoplankton. Fish, crustacean, cephalopod, and shellfish samples were identified to the lowest possible classification level in the laboratory and counted and weighed to the nearest 0.1 g wet weight. A Turner fluorometer was used to measure chlorophyll‐*a*, which was used to calculate the phytoplankton biomass (Parsons et al., 1984). Zooplankton samples were collected using plankton nets with a diameter of 37 cm and a mesh size of 112 μm throughout the water column, and the biomass was estimated on the basis of displaced volume, according to Ahlstrom and Thrailkill (Ahlstrom & Thrailkill, 1963). The mass of suspended particulate organic matter and deposited particulate organic matter were used to represent the mass of debris. The wet weight of suspended particulate organic matter was measured after 1 L of water was filtered by precombusted GF/F glass microfiber filter with a diameter of 0.7 μm. The wet weight of deposited particulate organic matter was measured after filtration through a 75‐μm mesh filter and pickling with 1 mol/L hydrochloric acid.

The P/B and Q/B data were based on the offshore sea area model of PRE in the northern South China Sea with similar latitude (Sun et al., 2016). The P/B and Q/B of fish and benthos were combined with the existing models of the northern South China Sea and part of China offshore with similar latitude (Chen et al., 2015; Duan, Li, Liu, Jiang, et al., 2009; Lee & Zhang, 2018; Rahman et al., 2019; Sun et al., 2016). The parameters of P/B or Q/B of phytoplankton were determined according to the existing models of the sea area around the PRE and the northern South China Sea (Chen et al., 2015; Sun et al., 2016) (Table S3), and the unit of P/B and Q/B was year^−1^.

Diet composition (Table S4) was developed from reports of stomach contents, FishBase (www.fishbase.org) and other relevant historical research materials for the different FGs (Chen et al., 2015; Duan, Li, Liu, Jiang, et al., 2009; Lee & Zhang, 2018; Rahman et al., 2019; Sun et al., 2016). Information for different groups was gathered from the same source when possible.

### 2.5 Summary statistics and comparisons with other ecosystem

A one‐way ANOVA was performed for the trophic levels calculated by all models. If there was no significant difference in the trophic levels of each model, the trophic levels of all FGs were averaged, and the regression simulation was performed with the historical reference trophic levels, or there is significant difference between the trophic levels of each model, regression is carried out between the trophic levels of each model and the historical reference trophic levels, respectively.

A number of statistics describing the ecosystem as a whole were used to determine the status of the PRE ecosystem. The total system throughput is the sum of all flows in the system, estimated as the sum of the four flow components: (1) the sum of all consumption; (2) the sum of all exports, that is, exported from the system by fisheries or buried in the sediments; (3) the sum of all respiration flows; and (4) the sum of all flows into detritus. The total system throughput represents the size of the system in terms of flows (Ulanowicz, 2012), and is important for comparisons of flow networks. The total net primary production is the sum of production by all the producers (i.e., phytoplankton and detritus) in the system. In a system, PPr/R is the ratio of total primary production (PPr) to total respiration (R). The ratio should be close to 1 in mature systems, which indicated the fixed energy and the cost of maintenance are approximately balanced. The ratio of PPr to total biomass (PPr/B) is expected to be a function of system maturity. The Finn's cycling index (FCI) is also correlated with system maturity, as a food chain is expected to be changed from the linear to web‐like one as the system becomes mature (Christensen et al., 2005; Odum & Barrett, 1971). The ‘Mature’ and ‘Stable’ systems generally display a high degree of recycling (Christensen, 1995). FCI shows the recycled part of the ecosystem's throughput. Recent work in this field shows that the index is strongly related to system maturity; in fact, diversity of flow and recycling (path length) are expected to increase with maturity. In order to diagnose the status of marine ranching ecosystems in the PRE, we selected coastal ecosystems of similar magnitude or the same latitude, such as other periods in the PRE, Beibu Gulf and Daya Bay (Chen et al., 2011, 2015; Duan, Li, Liu, Jiang, et al., 2009; Duan, Li, Liu, Moreau, et al., 2009; Rahman et al., 2019; Staebler et al., 2018; Wang et al., 2015), and the Bohai coastal ecosystem in northern China to compare food web properties which can evaluate the effects of AR construction and fishing moratorium in the marine pasture.

The concept of trophic level in the Ecopath model adopts the concept of fractional trophic level proposed by Odum (Odum & Helad, 1975). Each species is weighted according to the trophic level of its feed guild (usually assumed to be trophic level 1 for producer and detritus) and the proportion of its composition in the food. This concept of trophic level is also known as effective trophic level (ETL) (Leontief, 1965; Song, 2004), which can reflect the nutritional status of each FG in the ecosystem in more detail (Chen et al., 2008):

(1) $${\mathrm{ETL}}_{\mathrm{model}}=\frac{\sum_{i=1}^{N}\left(B_{i}*{\mathrm{TL}}_{i}\right)}{\sum_{i=1}^{N}B_{i}}$$

where ${\mathrm{ETL}}_{\mathrm{model}}$ is the trophic level of each model, *i* is the number of FGs, *N* is the total number of FGs, and ${\mathrm{ETL}}_{i}$ is the trophic level of FGs.

The keystone value of a given species is decided as a function of its biomass and the impact on the different elements of an ecosystem resulting from a small change to its own biomass (Libralato et al., 2006). Keystone species affect the communities of which they are part in a manner disproportionate to their abundance(Power et al., 1996). Keystone species strongly influence the abundances of other species and the ecosystem dynamic (Piraino et al., 2002). Therefore, defining the keystone groups that affect the premoratorium and postmoratorium or AR areas will help fishery resource management.

1. Linear regression was performed for the keystone shared by both models, and 95% confidence interval was plotted. The FGs not shared by both models could not be compared with each other.
2. All FGs within the confidence interval indicate a significant correlation between the two models. The function group located in the upper left region of the confidence interval indicates a significant influence on the *Y*‐axis, and the function group located in the lower right region of the confidence interval indicates a significant influence on the *X*‐axis.

## 3 RESULTS

### 3.1 Changes in ecosystem food web structure and energy transfer efficiency before and after fishing moratorium

**FIGURE 2:**
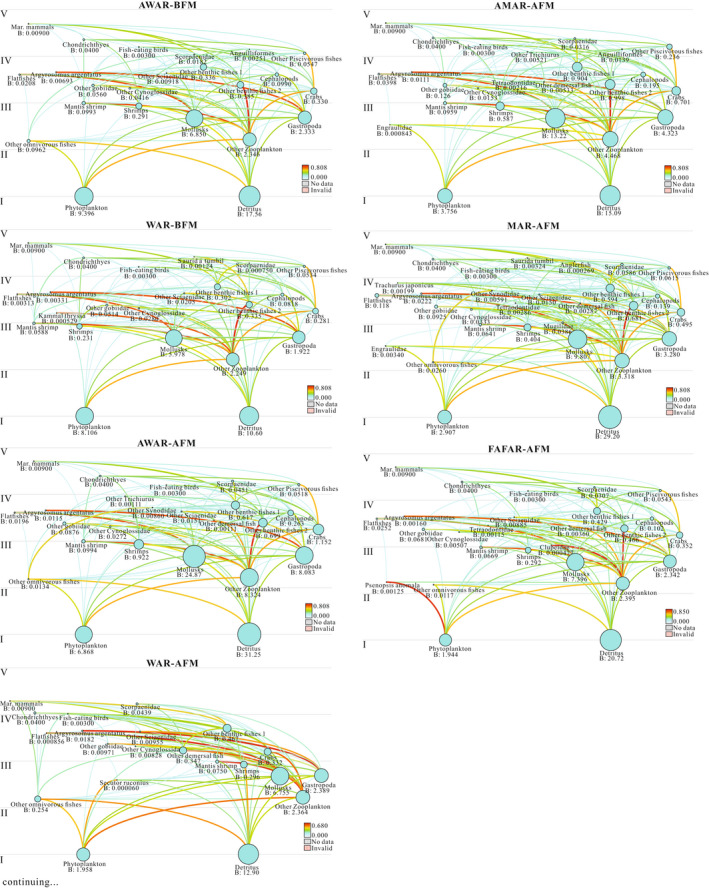
Flow diagrams representing food web structure in terms of functional groups and fractional trophic levels of seven different temporal and spatial models in the PRE marine ranching in 2020. The circles were distributed on the *Y*‐axis according to the nutrient level (I–V), and the size of the circles was proportional to the biomass of each group. Biomass is reported in tons per square kilometer (t km^−2^)

Ecological efficiency of all FGs is <1.00, indicating that the model is in equilibrium (Table S3). Phytoplankton and detritus constitute the main energy source for all models (Figure 2). The phytoplankton biomass mean values (8.11–9.40 t km^−2^) of the first two models before the fishing moratorium are significantly higher than the phytoplankton biomass mean values of the five models after the fishing moratorium (1.94–6.87 t km^−2^). The secondary trophic level (TL) consumers of fishery resources in the food web structure are the main FGs that transform the energy of primary producers. The omnivorous fish (including *Siganus fuscescens*, *Terapon jarbua*, and *Drepane punctata*) in the WAR‐AFM has a higher biomass (0.25 t km^−2^), which will further facilitate the transfer of energy from the bottom to up (Figure 2).

The energy transfer efficiency of the III and V‐TL was higher than that of the II and III‐TL before and after the fishing moratorium (Table 2), indicating that the low transformation efficiency of the low trophic level community hinders the material cycle and energy flow of the entire food web. Therefore, improving the transfer efficiency of low trophic levels will help to further promote the ecosystem stability and carrying capacity of the PRE marine ranching.

**TABLE 2:** The trophic transmission efficiency of seven different spatio‐temporal ecosystem models in the PRE marine ranching in 2020. ‘–’ means that no data are available

Trophic level	Transfer efficiency	AWAR‐BFM	WAR‐BFM	AWAR‐AFM	WAR‐AFM	AMAR‐AFM	MAR‐AFM	FAFAR‐AFM
I	Producer (%)	_	_	_	_	_	_	_
	Detritus (%)	_	_	_	_	_	_	_
	Total flow (%)	_	_	_	_	_	_	_
II	Producer (%)	13.11	11.88	12.83	12.94	13.24	13.19	13.58
	Detritus (%)	13.11	11.95	12.78	13.00	13.26	13.23	13.55
	Total flow (%)	13.11	11.91	12.81	12.96	13.25	13.21	13.57
III	Producer (%)	10.29	9.95	9.06	9.75	10.65	10.57	9.96
	Detritus (%)	10.70	10.40	9.40	9.80	11.06	10.98	10.40
	Total flow (%)	10.45	10.13	9.19	9.77	10.81	10.73	10.13
IV	Producer (%)	16.18	16.57	14.90	12.46	16.35	16.21	16.44
	Detritus (%)	16.18	16.55	14.97	12.14	16.36	16.23	16.44
	Total flow (%)	16.18	16.56	14.93	12.33	16.35	16.22	16.44
V	Producer (%)	15.94	16.22	15.27	7.69	15.95	16.10	16.28
	Detritus (%)	15.87	16.15	15.23	7.77	15.88	16.01	16.18
	Total flow (%)	15.91	16.19	15.25	7.72	15.92	16.06	16.24

### 3.2 Changes in food web structure and energy transfer efficiency in artificial and nonartificial reef ecosystems

Ecosystem models for artificial and nonartificial reef areas are also in equilibrium (Table S3) which can be used to compare food web structure and energy transfer efficiency. Phytoplankton and debris are also the primary energy sources for consumers in all models. The average biomass of phytoplankton and debris on ARs was not significantly different from that of nonartificial reefs. Miaowan and Wailingding had more fishery resource FGs than Wanshan, and their structures were more complex (Figure 2).

As far as the Wanshan is concerned, the energy transfer efficiency of the II and III‐TL of artificial fish reefs is slightly higher than that of nonartificial fish reefs (Table 2), indicating that artificial fish reefs can slightly promote the energy conversion efficiency of low trophic levels. However, the lack of management of II and III‐TL taxa may lead to its ineffective promotion effect. Therefore, the proliferation and release of low‐TL taxa can be appropriately increased and the reasonable fishing of such groups can be controlled. However, it has no significant impact on the energy transfer efficiency of artificial fishing reefs in other areas.

### 3.3 Ecosystem stability changes before and after the fishing moratorium

The mean values of ecosystem property indicators (consumption, respiration flow, total system throughput, and total biomass) after the fishing moratorium were significantly higher than those before the fishing moratorium. The PPr/R level before the fishing moratorium was significantly higher than that after the fishing moratorium, and the FCI value before the fishing moratorium was significantly lower than that after the fishing moratorium. It shows that the overall metabolism of the ecosystem and the biomass production efficiency is high after the fishing moratorium, which can improve the overall status of the ecosystem and help maintain the stability of the ecosystem (Table 3).

**TABLE 3:** Comparative analysis of selected ecosystem attributes of seven different spatio‐temporal models in the PRE marine ranching in 2020. ‘–’ indicates no data

Type	Statistics and flows					Community energetics		Organic matter cycling
	Sum of all consumption	Sum of all respiratory flows	Total system throughput	Total biomass (excluding detritus)	Transfer efficiencies from primary producers	Total primary production/total respiration	Shannon diversity index	Finn’s cycling index
AWAR‐BFM^a^	830.22	441.52	5047.35	22.82	12.97	5.00	1.59	4.13
WAR‐BFM^a^	776.00	410.77	4402.66	19.76	12.51	4.64	1.56	4.58
AWAR‐AFM^a^	2929.35	1559.39	5454.96	52.23	12.01	1.04	1.55	21.16
WAR‐AFM^a^	837.58	445.51	1557.20	15.38	11.63	1.03	1.71	20.99
AMAR‐AFM^a^	1582.45	841.51	2967.11	29.78	13.21	1.05	1.73	20.51
MAR‐AFM^a^	1174.96	624.98	2258.79	22.23	13.12	1.09	1.75	19.43
FAFAR‐AFM^a^	853.56	454.81	1560.76	16.05	13.05	1.00	1.69	21.55
PRE^b^	4969.93	3139.81	15243.00	265.88	–	2.87	–	–
sNoSe^c^	6227.00	1960.20	12232.40	556.50	–	1.10	–	–
MDNS^c^	6150.50	1924.70	12048.70	551.80	–	1.12	–	–
Global min – max^c^	–	–	500–170,000	17–3900	–	–	–	–
BGM‐1960s^d^	5196.84	3163.13	10557.00	110.79	–	1.01	–	18.60
BGM‐1990s^d^	4037.87	2391.70	13753.00	96.09	–	2.18	–	9.73
PRE‐1981^e^	1377.53	587.34	4799.00	71.53	–	2.86	–	9.21
PRE‐1998^e^	285.22	128.48	1764.00	32.93	–	5.83	–	2.72
PRE‐2008^f^	273.04	122.72	2311.86	39.90	–	8.36	–	2.22
DYB^g^	2211.50	1296.60	11409.20	55.50	10.30	3.50	–	2.17
Bohai^h^	691.43	411.31	10074.20	28.54	11.18	11.71	1.13	0.89
Units	t km^−2^ year^−1^	t km^−2^ year^−1^	t km^−2^ year^−1^	t km^−2^	%	dimensionless	dimensionless	dimensionless

^a^ This study.

^b^ Pearl River Estuary coastal ecosystem 1997–1999 (Duan, Li, Liu, Jiang, et al., 2009).

^c^ southern North Sea ecosystem 1991 (Staebler et al., 2018).

^d^ Beibu Gulf model (Chen et al., 2011).

^e^ Coastal ecosystem of the Pearl River Estuary (Duan, Li, Liu, Moreau, et al., 2009).

^f^ PRE coastal ecosystem 1998,2008 (Wang et al., 2015).

^g^ Daya Bay ecosystem 2011 (Chen et al., 2015).

^h^ Bohai Sea ecosystem 2016 (Rahman et al., 2019).

### 3.4 Ecosystem stability changes between artificial and nonartificial reefs

The average value of the ecosystem attribute indicators (consumption, respiration flow, total system throughput, and total biomass) of ARs is lower than that of nonartificial reef areas, which is inconsistent with AR construction helping to improve fisheries. It shows that the construction of ARs in this area has no significant effect on the improvement of ecosystem production efficiency, and there is no significant difference between the ecosystem stability indicators PPr/R and FCI levels between ARs and nonartificial reefs. However, the system is close to a steady state, which may be the effect of the fishing moratorium (Table 3).

### 3.5 Keystone communities affecting different ecosystems

**FIGURE 3:**
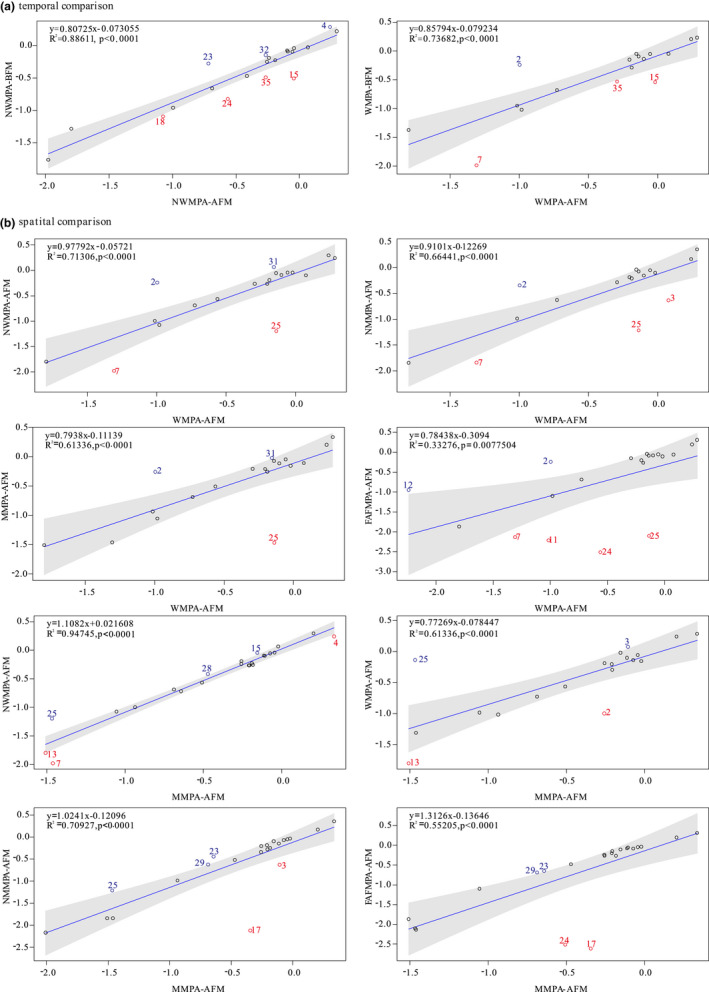
Comparison of food web keystone index between seven different spatio‐temporal ecosystem models in the PRE marine ranching in 2020. Numbers refer to functional group codes. The functional groups showing relatively equal values in both food webs are black colored. In both food webs, a higher criticality index in one of the food webs is blue (upper left half) or red (lower right half)

Keystone groups have strong influence on the abundance of other species and their own abundance (Libralato et al., 2006; Power et al., 1996). It is important to identify keystone groups to glean further insight in the ecosystem structure. The keystone species are those groups with indicator values close to or greater than zero. The keystone index in the model was analyzed by linear fitting. In terms of time, the results showed that keystone species affecting the Wanshan AR are 2 (Chondrichthyes) before the fishing moratorium, and 7, 15, and 35 (*A. argentatus*, Scorpaenidae fishes, and Phytoplankton) after the fishing moratorium. In addition, groups 4, 23, and 32 (flatfishes, other piscivorous fishes including grouper and tapfish, and other Bivalvia) affected the adjacent Wanshan ARs before the fishing moratorium, and groups 15, 18, 24, and 35 (Scorpaenidae fish, other Sciaenidae, other omnivorous fishes, and Phytoplankton) affected the adjacent Wanshan ARs after the fishing moratorium. The key species affecting the same area were different in different periods. In terms of space, we focused on the differences of keystone species in the two ARs with other regions. The keystone species affecting the Wanshan AR area was 25(other demersal fish), and the species affecting Miaowan AR area was 17(Tetraodontidae) which is reflected in only two diagrams, indicating that there were differences in the key species affecting different regions (Figure 3).

## 4 DISCUSSION

### 4.1 Effect of fishing moratorium and artificial reef construction

**FIGURE 4:**
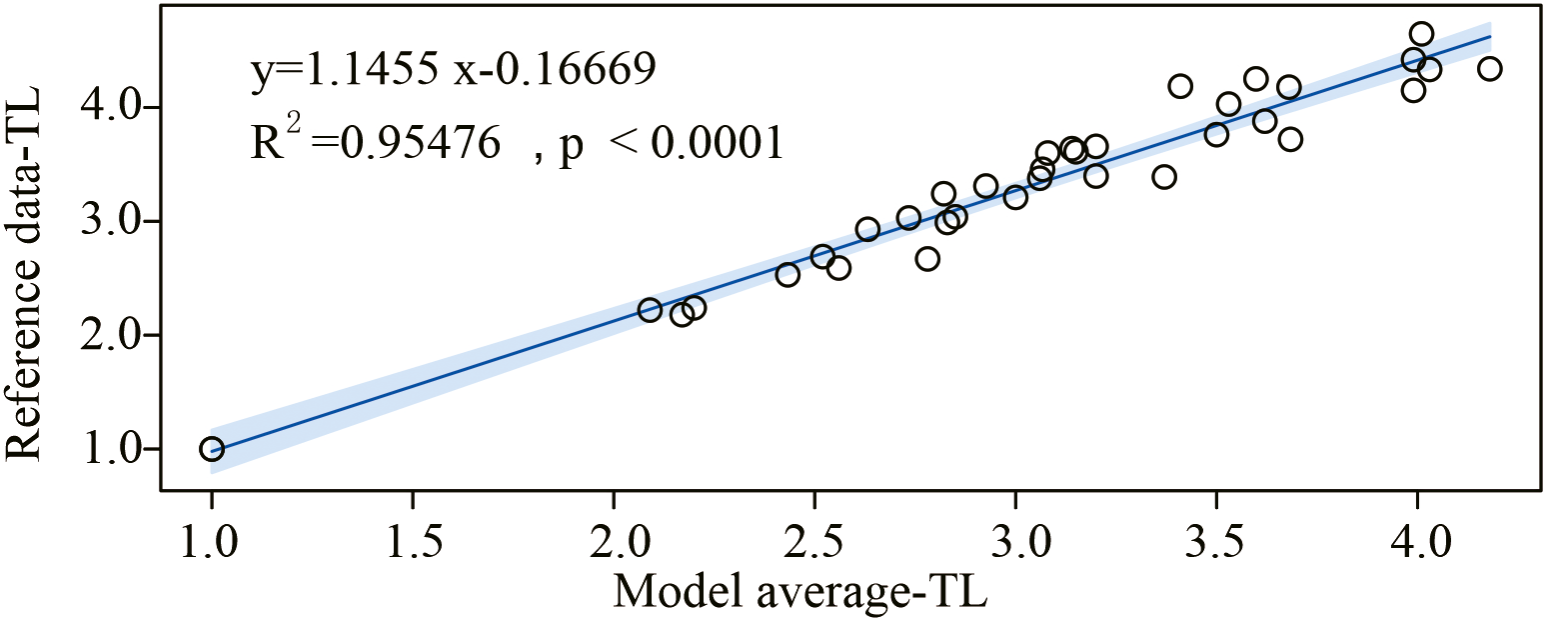
Linear regression between model average‐TL and historical references‐TL in the PRE marine ranching in 2020

This research is based on ARs placed and fishing moratorium in the traditional fishing grounds of the PRE to restore degraded natural habitats and fisheries. Previous groups have used the EWE model to study the food web structure in the northern South China Sea and offshore China (Chen et al., 2015; Duan, Li, Liu, Jiang, et al., 2009; Lee & Zhang, 2018; Rahman et al., 2019; Sun et al., 2016). TL of the model in this study and TL of previous studies resulted have a good linear regression fitting (Figure 4), indicating that the estimation results of the model are within the range of actual measurement indicators, which verifies the accuracy of the model.

Comparing the trophic levels before and after the fishing moratorium, the ETLs of WAR‐BFM and AWAR‐BFM were 1.65 and 1.56, and WAR‐AFM and AWAR‐AFM were 1.79 and 1.90 indicating that fishing leads to a slight decline in trophic levels in the system, consistent with the trend of “fishing down the food web” as documented by Pauly et al. (1998). Previous studies have shown that the catches in the PRE ecosystem mainly come from II and III‐TL (Duan, Li, Liu, Moreau, et al., 2009), and it was speculated that the biomass of II and III‐TL decreased before the fishing moratorium, resulting in the decrease of the average conversion efficiency of two trophic levels and hindered the material circulation (Table 2, Figure 2). Therefore, the rational harvesting of these two trophic levels of organisms will promote the material cycle and energy flow of the ecosystem.

Succession is considered to be an orderly process of community development toward a mature stage, and maturity is the last state in the succession process (Odum, 1969). The differences in the system maturity of the seven models are mainly reflected in the pre‐ and postfishing moratorium, the postfishing moratorium ecosystem is more mature than the prefishing moratorium system, which is presumably due to overfishing. The ecosystem before the fishing moratorium is in a medium–low stage of development (Coll et al., 2006, 2007); the PPr/R value of the PRE in 1998 and 2008 was higher than the PPr/R value of the northern South China Sea or the PRE in 1960, 1981, and 1990s. However, the fishing moratorium has been implemented in the South China Sea since 1999, indicating that the stabilization of entire ecosystem has not significantly improved from the moratorium alone. There are two possible reasons for this improvement: one is that after the temporary fishing moratorium, fishermen increase their fishing efforts on fishery resources; the other is that the temporary fishing moratorium only has a recovery effect on fishery resources with a short life cycle. However, in 2011 and 2012, ARs were placed in Miaowan and Wanshan of the PRE (internal data), and the PPr/R values of all models in this study were lower than those of the PRE in 1998 and 2008. It is speculated that the construction of a marine ranching has a positive effect on the restoration of the ecosystem in this area.

Existing research results have shown that fishing exploitation impacts on the keystone predators determines the obvious anthropogenic changes in the food web, which in turn affects the structure and function of the marine ecosystem (Baum & Worm, 2009; Pauly et al., 1998). Therefore, in the critical ecological process of maintaining the entire community, some processes are driven by keystone species which must be identified and used by the “conservation biologist’s toolbox” (Power et al., 1996). Keystone groups play an important ecological function, including maintaining the food web structure of their community (Perry, 2010). Thus, identifying and protecting keystone groups may be the only long‐term solution for protecting a "working ecosystem rather than a collection of charismatic species" (Ferenc et al., 2009; Perry, 2010). In addition, finding keystone species that influence temporal or spatial differences will be a major challenge (Paine, 1995; Power & Mills, 1995). Therefore, consideration of spatiotemporal scales is an important factor in keystone species identification. In this study, our results align with the result of Power et al. and Piraino et al. that keystone species are not straightforwardly predicable in terms of timing (Piraino et al., 2002; Power et al., 1996). Our results also shows that the keystone species affecting the Wanshan AR area is mainly *Nemipterus japonicus*, and the keystone species affecting the Miaowan AR area may be *Gastrophysus spadiceus*. However, the species that are proliferated and released in the PRE are mainly snappers and grouper (Liu et al., 2019), which are weaker than *N. japonicus* and *G. spadiceus* in maintaining the stability of the ecosystem in this area. In addition, the loss of functional roles can lead to a decreased ecological stability, and ecosystems can become both less resilient to natural disturbance and less resistant to invasion by exotic species (Stallings, 2009). *Nemipterus japonicus* and *G. spadiceus* are the main commercial species in Wanshan and Miaowan, and the two species are located in III‐TL, effectively transferring energy to higher trophic levels through feeding on benthic organisms, phytoplankton, and benthic debris. Therefore, these groups should be focused on Wanshan ranching management. This not only contributes to the development of effective species‐level priority conservation strategies but also to a better understanding of ecosystem functions and processes (Clemente et al., 2010; Ferenc et al., 2009).

### 4.2 Management of fishing moratorium and artificial reef construction and suggestions for future studies

Wang et al. (2015) postulated that the fishing moratorium has achieved the purpose of protecting the juvenile fish communities, as the period coincides with the growth period of most juvenile fish. In previous studies (Cohen & Foale, 2013; Foale & Manele, 2004; Russ & Alcala, 1998), suspension of fishing was considered more suitable for short‐lived and fast‐growing species rather than long‐lived and slow‐growing species. However, habitat degradation has been well documented in the United States, China, and globally (Beck et al., 2011; Quan et al., 2006; Shen et al., 2011; Wilberg et al., 2011), and is another factor contributing to the decline of fishery resources. Artificial reefs play an important role in supporting fish production, and the use of ecosystem modeling to study the spatio‐temporal changes in ecosystem attributes and food web structure is of great significance for assessing ecosystem health and managing fishery production (Kremer & Nixon, 2012). Therefore, the fishing moratorium should be combined with the construction of ARs to be more conducive to the recovery of fishery resources.

Although the ecosystem model can provide a good plan for studying the ecosystem, it is difficult to obtain the model parameters of the specific research area. Most of the research parameters come from similar models. Therefore, it needs to be combined with new detection technologies such as stable isotopes and fatty acids to optimize the parameters. Furthermore, in the context of the decline of global fishery resources, it is important to use inter‐regional model comparisons to identify keystone communities for recovery. At the same time, managers can also propose more reasonable proliferation, release, and fishing strategies based on the research results to help the restoration of the ecosystem.

## 5 CONCLUSION

Ecological simulation is an effective way to study aquatic ecosystems. Models can be used to improve our understanding of the fishery resource predator–forage interaction in the system. In addition, ecological functions can also be integrated according to the energy flow of the food web. The conclusions of this study are as follows:

1. The nutritional status and development stages of seven different spatio‐temporal models found that the ecosystem is more stable after the fishing moratorium than before the fishing moratorium. Rational development and utilization of II and III‐TL organisms can reduce the impact of fishing on the food web.
2. Compared with previous studies, the fishing moratorium system cannot guarantee the sustainability of the restoration of the entire ecosystem. The construction of artificial fish reefs in marine ranching is conducive to improving the stability of the ecosystem structure, but the energy conversion efficiency has not been improved.
3. The ranking results of the keystone index of different models showed that *N. japonicus* and *G. spadiceus* are keystone species that distinguish the Wanshan and Miaowan ARs from other areas. However, the species that are proliferated and released in the PRE are mainly snappers and grouper, which are weaker than *N. japonicus* and *G. spadiceus* in maintaining the stability of the ecosystem in this area. In addition, the loss of functional roles can lead to a decreased ecological stability and ecosystems can become both less resilient to natural disturbance and less resistant to invasion by exotic species. *Nemipterus japonicus* and *G. spadiceus* are the main commercial species in Wanshan and Miaowan, and the two species are located in TL‐Ⅲ, effectively transferring energy to higher trophic levels through feeding on benthic organisms, phytoplankton, and benthic debris. Therefore, reasonable fishing should also be considered. Further quantitative studies on its reasonable catches are required in the future.

## AUTHOR CONTRIBUTIONS

**Peng Xu:** Conceptualization (lead); data curation (lead); formal analysis (lead); investigation (lead); methodology (lead); visualization (lead); writing – original draft (lead); writing – review and editing (lead). **Mujiao Xie:** Data curation (supporting); investigation (supporting); visualization (supporting). **Weiguo Zhou:** Data curation (equal); investigation (supporting); visualization (supporting). **Dewen Ding:** Conceptualization (lead); funding acquisition (supporting); resources (lead). **Anning Suo:** Conceptualization (lead); funding acquisition (supporting); resources (lead).

## Supporting information

Table S1‐S5

## Data Availability

Data supporting this manuscript are presented in the main text and in appendices provided.
