# Supplementary material for: Temporal and spatial comparison of food web structure in marine pastures in the Pearl River Estuary: Implications for sustainable fisheries management
Source: Ecol Evol. 2022 May 13;12(5):e8903. doi: 10.1002/ece3.8903 (PMC9102640; doi:10.1002/ece3.8903)
Supplement: Supplementary file 1 — Table S1‐S5 [file ECE3-12-e8903-s001.docx]

**SUPPLEMENTARY INFORMATION**

**Table S1.** **Classification** **for all function groups of 7 different spatio-temporal models in the PRE marine pasture in 2020.** '-' indicates no data.

| Group number | Group name | Group description | Species composition | | | | | | |
| --- | --- | --- | --- | --- | --- | --- | --- | --- | --- |
|  |  |  | AWAR-BFM | WAR-BFM | AWAR-AFM | WAR-AFM | AMAR-AFM | MAR-AFM | FAFAR-AFM |
| 1 | Marine mammals | Delphinidae, Neophocaena, Dugong dugon, etc. | Mar. mammals | Mar. mammals | Mar. mammals | Mar. mammals | Mar. mammals | Mar. mammals | Mar. mammals |
| 2 | Chondrichthyes | Scoliodon sorrakowah, Dasyatis  bennetti, Dasyatis zugei, etc. | Chondrichthyes | Chondrichthyes | Chondrichthyes | Chondrichthyes | Chondrichthyes | Chondrichthyes | Chondrichthyes |
| 3 | Fish-eating birds | Charadriidae, Apodidiae, Jacanidae,  Laridae, etc. | Fish-eating birds | Fish-eating birds | Fish-eating birds | Fish-eating birds | Fish-eating birds | Fish-eating birds | Fish-eating birds |
| 4 | Flatfishes | *Pseudorhombus cinnamoneus*, *Kareius bicoloratus*, *Pseudorhombus cinnamomeus*, *Pseudorhombus oligodon*, *Solea ovata*, *Zebrias quagga*, Arnoglossus tenuis, *Aseraggodes kobensis*, *Crossorhombus kobensis*, *Brachypleura novaezeelandiae*, etc. | *Pseudorhombus cinnamomeus*, *Pseudorhombus oligodon*, *Solea ovata*, *Zebrias quagga* | *Pseudorhombus oligodon*, *Solea ovata* | *Arnoglossus tenuis*, *Pseudorhombus cinnamomeus*, *Solea ovata* | *Arnoglossus tenuis* | *Aseraggodes kobensis*, *Solea ovata*, *Crossorhombus kobensis*, *Arnoglossus tenuis*, *Pseudorhombus cinnamomeus*, *Pseudorhombus oligodon*, *Brachypleura novaezeelandiae* | *Zebrias quagga*, *Aseraggodes kobensis*, *Solea ovata*, *Crossorhombus kobensis*, *Arnoglossus tenuis*, *Pseudorhombus cinnamomeus*, *Pseudorhombus oligodon*, *Brachypleura novaezeelandiae* | *Solea ovata*, *Arnoglossus tenuis* |
| 5 | Saurida tumbil | single species | - | *Saurida tumbil* | - | - | - | *Saurida tumbil* | - |
| 6 | Trachurus japonicus | single species | - | - | - | - | - | *Trachurus japonicus* | - |
| 7 | Argyrosomus argentatus | single species | Argyrosomus argentatus | *Argyrosomus argentatus* | *Argyrosomus argentatus* | *Argyrosomus argentatus* | *Argyrosomus argentatus* | *Argyrosomus argentatus* | *Argyrosomus argentatus* |
| 8 | Psenopsis anomala | single species | - | - | - | - | - | - | *Psenopsis anomala* |
| 9 | Thryssa kammalensis | single species | - | *Thryssa kammalensis* | - | - | - | - | - |
| 10 | Anglerfish | *Lophius litulon*, *Halieutaea stellata*, etc. | - | - | - | - | - | Halieutaea stellata | - |
| 11 | Other gobiidae | *Acanthogobius hasta*, *Cryptocentrus filifer*, *Trypauchen vagina*, *Parachaeturichthys polynema*, *Oxyurichthys tentacularis*, etc. | *Cryptocentrus filifer*, *Trypauchen vagina* | *Cryptocentrus filifer*, *Trypauchen vagina*, *Parachaeturichthys polynema* | *Cryptocentrus filifer*, *Trypauchen vagina*, *Parachaeturichthys polynema*, *Oxyurichthys tentacularis* | *Trypauchen vagina*, *Parachaeturichthys polynema* | *Trypauchen vagina*, *Oxyurichthys tentacularis* | *Oxyurichthys tentacularis* | *Acanthogobius hasta* |
| 12 | Secutor ruconius | single species | - | - | - | *Secutor ruconius* | - | - | - |
| 13 | Other Cynoglossidae | *Cynoglossus oligolepis*, *Paraplagusia japonica*, *Cynoglossus abbreviatus*, *Cynoglossus puncticeps*, *Cynoglossus macrolepidotus*, *Cynoglossus lineolatus*, etc. | *Cynoglossus abbreviatus*, *Cynoglossus puncticeps*, *Cynoglossus macrolepidotus* | *Cynoglossus abbreviatus*, *Cynoglossus puncticeps*, *Cynoglossus macrolepidotus* | *Cynoglossus abbreviatus*, *Cynoglossus puncticeps*, *Cynoglossus macrolepidotus* | *Cynoglossus macrolepidotus* | *Cynoglossus macrolepidotus*,  *Cynoglossus lineolatus* | *Cynoglossus abbreviatus*, *Cynoglossus puncticeps*, *Cynoglossus macrolepidotus*, *Cynoglossus lineolatus* | *Cynoglossus puncticeps*,  *Cynoglossus macrolepidotus* |
| 14 | Other Trichiurus | *Trichiurus lepturus*, *Trichiurus brevis*, *Lepturacanthus savala*, etc. | - | - | *Lepturacanthus savala* | - | *Lepturacanthus savala* | - | - |
| 15 | Scorpaenidae | *Sebastiscus marmoratus*, *Parascorpaena picta*, *Trachicephalus uranoscopa*, *Platycephalus spp*., *Onigocia macrolepis*, *Grammoplites scaber*, *Lepidotrigla alata*, *Platycephalus indicus*, *Minous inermis*, etc. | *Trachicephalus uranoscopa*, *Platycephalus spp*., *Onigocia macrolepis*, *Grammoplites scaber*, *Lepidotrigla alata* | *Trachicephalus uranoscopa* | *Platycephalus spp*., *Platycephalus indicus*, *Trachicephalus uranoscopa*, *Grammoplites scaber*, *Lepidotrigla alata* | *Trachicephalus uranoscopa*,  *Platycephalus spp*., *Grammoplites scaber* | *Platycephalus spp*., *Platycephalus indicus*, *Minous inermis*, *Lepidotrigla alata* | *Platycephalus spp*., *Platycephalus indicus*, *Minous inermis*, *Lepidotrigla alata* | *Platycephalus spp*., *Platycephalus indicus*, *Grammoplites scaber*, *Trachicephalus uranoscopa* |
| 16 | Other Synodidae | *Saurida elongata*, *Saurida undosquamis*, etc. | - | - | *Saurida elongata* | - | - | *Saurida undosquamis* | - |
| 17 | Tetraodontidae | *Navodon xanthopterus*, *Gastrophysus spadiceu*s, *Fugu xanthopterus*, *Paramonacanthus nipponensis*, etc. | - | - | - | - | *Gastrophysus spadiceus* | *Gastrophysus spadiceus* | *Gastrophysus spadiceus* |
| 18 | Other Sciaenidae | *Otolithes argenteus*, *Umbrina russelli*, *Argyrosomus argentatus*, *Chrysochir aureus*, etc. | *Argyrosomus argentatus* | *Argyrosomus argentatus*, *Chrysochir aureus* | *Argyrosomus argentatus* | *Argyrosomus argentatus* | - | *Argyrosomus argentatus* | *Argyrosomus argentatus* |
| 19 | Mugilidae | *Osteomugil ophuyseni*, *Polynemus sextarius*, etc. | - | - | - | - | - | *Polynemus sextarius* | - |
| 20 | Clupeidae | *Sardinella clupeoides*, *Sardinella nymphaea*, etc | - | - | - | - | - | - | *Sardinella zunas* |
| 21 | Engraulidae | *Sardinella nymphaea*, *Setipinna tenuifilis*, *Thrissa setirostris*, *Thryssa hamiltonii*, etc. | - | - | - | - | *Setipinna tenuifilis* | *Setipinna tenuifilis*, *Thrissa setirostris*, *Thryssa hamiltonii* | - |
| 22 | Anguilliformes | *Conger myriaster*, *Dysomma anguillaris*, *Pisodonophis cancrivorus*, *Strophidon sathete*, etc. | *Dysomma anguillaris* | - | - | - | *Pisodonophis cancrivorus*, *Strophidon sathete* | - | - |
| 23 | Other Piscivorous fishes | Scombermorus, Epinephelus, Aulopiformes, etc. | *Epinephelus awoara* | *Epinephelus bleekeri* | *Harpadon nehereus* | - | *Epinephelus awoara*,  *Harpadon nehereus* | *Terapon theraps*, *Pelates quadrilineatus*, *Harpadon nehereus* | *Harpadon nehereus* |
| 24 | Other omnivorous fishes | *Siganus oramin*, *Siganus fuscescens*, *Therapon jarbua*, *Drepane punctata*, etc. | *Siganus fuscescens*, *Drepane punctata* | - | Drepane punctata | Siganus fuscescens | - | Therapon jarbua | Drepane punctata |
| 25 | Other demersal fish | Labridae, Scaridae, Nemipterus, etc. | - | - | *Nemipterus Japonicus* | *Nemipterus Japonicus* | *Nemipterus Japonicus* | *Nemipterus Japonicus* | *Nemipterus Japonicus* |
| 26 | Other benthic fishes 1 | *Raja porosa*, *Liparis tanakae*, *Jaydia lineata*, *Apogon quadrifasciatus*, *Apogon ellioti*, *Hexagrammos otakii*, *Takifugu vermicularis*, etc. | Apogon quadrifasciatus | Jaydia lineata,  Apogon quadrifasciatus, Apogon ellioti, | Apogon quadrifasciatus | Apogon quadrifasciatus | Jaydia lineata,  Apogon quadrifasciatus, | Jaydia lineata, Apogon quadrifasciatus, Apogon ellioti, | Apogon quadrifasciatus |
| 27 | Other benthic fishes 2 | *Parapercis sexfasciata*, *Callionymus koreanus*, *Sillago japonica*, *Sillago sihama*, *Uranoscopus oligolepis*, *Arius sinensis*, *Hapaloyenys mucronatus*, *Upeneus japonicus*, *Parapercissex fasciata*, *Champsodon snyderi*, etc. | *Parapercis sexfasciata* | *Callionymus koreanus*,  *Sillago japonica* | *Callionymus koreanus* | - | *Callionymus koreanus*,  *Sillago japonica* | *Sillago sihama*, *Callionymus koreanus*,  *Sillago japonica*, *Uranoscopus oligolepis* | *Callionymus koreanus*, *Arius sinensis* |
| 28 | Cephalopods | *Loligo edulis*, *Loligo duvaucelii*, *Loligo chinensis*, *Octopus ocellatus*, *Euprymna berryi*, *Sepiella maindroni*, etc. | *Euprymna berryi* | *Euprymna berryi* | *Euprymna berryi*, *Octopus ocellatus* | - | *Sepiella maindroni*, *Loligo duvaucelii* | *Sepiella maindroni*, *Loligo duvaucelii*,  *Octopus ocellatus* | - |
| 29 | Mantis shrimp | *Oratosquilla woodmasoni*, *Harpiosquilla harpax*,  *Oratosquilla kempi*, *Squilla nepa*, *Oratosquilla oratoria*, etc. | *Harpiosquilla harpax*, *Oratosquilla kempi*, *Squilla nepa*, *Oratosquilla oratoria* | *Harpiosquilla harpax*, *Oratosquilla kempi*, *Squilla nepa*, *Oratosquilla oratoria*, | *Oratosquilla woodmasoni*, *Harpiosquilla harpax*,  *Oratosquilla kempi*, *Squilla nepa*, *Oratosquilla oratoria* | *Harpiosquilla harpax*, *Oratosquilla kempi*, *Squilla nepa*, *Oratosquilla oratoria* | *Harpiosquilla harpax*, *Oratosquilla kempi*, *Oratosquilla oratoria* | *Oratosquilla woodmasoni*, *Harpiosquilla harpax*, *Oratosquilla kempi* | *Harpiosquilla harpax*, *Oratosquilla kempi Squilla nepa*, *Oratosquilla oratoria* |
| 30 | Shrimps | *Banana prawn*, *Parapenaeopsis hungerfordi*, *Metapenaeus affinis*, *Metapenaeus ensis*, *Penaeus japonicus*, *Metapenaeus joyneri*, *Metapenaeopsis palmensis*, *Trachypenaeus curvirostris*, *Solenocera crassicornis*, *Penaeus penicillatus*, *Alpheus distinguendus*, *Parapenaeopsis tenella*, *Parapenaeopsis hardwickii*, *Metapenaeopsis barbata*, etc. | *Banana prawn*, *Parapenaeopsis hungerfordi*, *Metapenaeus affinis*, *Metapenaeus ensis*, *Penaeus japonicus*, *Metapenaeus joyneri*, *Metapenaeopsis palmensis*, *Trachypenaeus curvirostris*, *Solenocera crassicornis* | *Banana prawn*, *Parapenaeopsis hungerfordi*, *Metapenaeus affinis*, *Metapenaeus ensis*, *Penaeus japonicus*, *Penaeus penicillatus*, *Metapenaeus joyneri*, *Metapenaeopsis palmensis*, *Trachypenaeus curvirostris*, *Solenocera crassicornis*, *Alpheus distinguendus* | *Parapenaeopsis hungerfordi*, *Metapenaeus affinis*, *Parapenaeopsis tenella*, *Parapenaeopsis hardwickii*, *Metapenaeus joyneri*, *Metapenaeopsis barbata*, *Trachypenaeus curvirostris*, *Alpheus distinguendus*, *Solenocera crassicornis* | *Parapenaeopsis hungerfordi*,  *Metapenaeus affinis*, *Penaeus penicillatus*, *Parapenaeopsis tenella*, *Metapenaeus joyneri*, *Solenocera crassicornis* | *Parapenaeopsis hungerfordi*,  *Metapenaeus affinis*, *Banana prawn*, *Penaeus penicillatus*, *Metapenaeopsis barbata*, *Trachypenaeus curvirostris*, *Solenocera crassicornis* | *Metapenaeus affinis*, *Penaeus penicillatus*, *Metapenaeopsis barbata*, *Trachypenaeus curvirostris* | *Parapenaeopsis hungerfordi*,  *Metapenaeus affinis*, *Banana prawn*, *Penaeus penicillatus*, *Parapenaeopsis hardwickii*, *Metapenaeus joyneri*, *Metapenaeopsis barbata*, *Trachypenaeus curvirostris*, *Alpheus distinguendus* |
| 31 | Crabs | *Scalopidia spinosipes*, *Eucrate alcocki*, *Eucrate crenata*, *Charybdis feriatus*, *Portunus pelagicus*, *Portunus hastatoides*, *Portunus gracilimanus*, *Charybdis truncatus*, *Charybdis vadorum*, *Charybdis variegata*, *Charybdis bimaculata*, *Dorippe japonica*, *Arcania heptacantha*, *Parthenope validus*, *Charybdis acuta*, *Podophthalmus vigil*, *Portunus sanguinolentus*, *Scylla serrata*, *Calappa philargius*, *Charybdis lucifera*, *Charybdis japonica*, *Carcinoplax purpurea*, Charybdis miles, *Liagore rubromaculata, Demania scaberrima*, *Leucosia rhomboidalis*, *Ixa edwardsii*, *Cryptopodia fronicata*, etc. | *Scalopidia spinosipes*,  *Eucrate alcocki*, *Eucrate crenata*, *Charybdis feriatus*, *Portunus pelagicus*, *Portunus hastatoides*, *Portunus gracilimanus*, *Charybdis truncatus*, *Charybdis vadorum*, *Charybdis variegata*, *Charybdis bimaculata*, *Dorippe japonica*, *Arcania heptacantha*, *Parthenope validus* | *Scalopidia spinosipes*,  *Eucrate alcocki*, *Eucrate crenata*, *Charybdis feriatus*, *Portunus hastatoides*, *Charybdis truncatus*, *Charybdis vadorum*, *Charybdis variegata*, *Charybdis acuta*, *Charybdis bimaculata*, *Dorippe japonica*, *Arcania heptacantha* | *Eucrate crenata*, *Eucrate alcocki*, *Scalopidia spinosipes*, *Charybdis variegata*, *Charybdis vadorum*, *Charybdis truncatus*, *Podophthalmus vigil*, *Portunus sanguinolentus*, *Portunus hastatoides*, *Portunus gracilimanus*, *Charybdis feriatus*, *Scylla serrata*, *Calappa philargius* | *Eucrate alcocki*, *Scalopidia spinosipes*, *Charybdis variegata*, *Charybdis vadorum*, *Charybdis lucifera*, *Charybdis truncatus*, *Podophthalmus vigil*, *Dorippe japonica*, *Portunus sanguinolentus*, *Portunus hastatoides*, *Portunus gracilimanus*, *Charybdis feriatus*, *Charybdis japonica* | *Carcinoplax purpurea*, *Eucrate alcocki*, *Arcania heptacantha*, *Charybdis miles*, *Charybdis truncatus*, *Podophthalmus vigil*, *Dorippe japonica*, *Liagore rubromaculata*, *Demania scaberrima*, *Portunus hastatoides*, *Portunus gracilimanus*, *Calappa philargius* | *Eucrate crenata*, *Leucosia rhomboidalis*, *Arcania heptacantha*, *Ixa edwardsii*, *Charybdis truncatus*, *Portunus sanguinolentus*, *Portunus hastatoides*, *Portunus gracilimanus*, *Cryptopodia fronicata*, *Calappa philargius* | *Eucrate crenata*, *Eucrate alcocki*, *Scalopidia spinosipes*, *Charybdis truncatus*, *Podophthalmus vigil*, *Dorippe japonica*, *Portunus hastatoides*, *Portunus gracilimanus*, *Charybdis feriatus* |
| 32 | Other mollusks | Bivalvia | Bivalvia | Bivalvia | *Vepricardium coronatum*, *Placamen calophylla*, *Scapharca broughtonii*, *Scapharca subcrenata* | *Vepricardium coronatum*,  *Placamen calophylla*, *Scapharca subcrenata*, *Paphia undulata* | *Vepricardium coronatum*,  *Placamen calophylla*, *Scapharca subcrenata*, *Amussium Pleuronectes*, *Solidicorbula erythrodon* | *Vepricardium coronatum*,  *Placamen calophylla*, *Amussium Pleuronectes* | *Vepricardium coronatum*, *Placamen calophylla*, *Scapharca broughtonii*, *Scapharca subcrenata*, *Paphia undulata* |
| 33 | Other Zooplankton | Copepods, Chaetognatha, Cladocera, etc. | Other Zooplankton | Other Zooplankton | Other Zooplankton | Other Zooplankton | Other Zooplankton | Other Zooplankton | Other Zooplankton |
| 34 | Gastropoda | Haliotis, Trochidae, Bursidae, etc. | Gastropoda | Gastropoda | *Bursa rana*, *Callista erycina*, *Turritella terebra*, *Calyptraea morbida*, *Natica spadicea*, *Murex trapa*, *Rapana bezona*, *Rapana venosa*, *Turricula nelliae*, *Turricula javana*, *Nassarius siquijorensis* | *Bursa rana*, *Callista erycina*, *Turritella terebra*, *Polynices mammata*, *Sinum planulatum*, *Semicassis bisulcatum*, *Murex trapa*, *Turricula nelliae*, *Turricula javana*, *Nassarius siquijorensis* | *Bursa rana*, *Callista erycina*, *Tonna olearium*, *Turricula javana*, *Nassarius siquijorensis* | *Bursa rana*, *Callista erycina*, *Murex trapa* | *Bursa rana*, *Turritella terebra*, *Murex trapa*, *Turricula nelliae*, *Babylonia areolata* |
| 35 | Phytoplankton | Bacillariophyta, Pyrrophyta, Cyanophyta, Chrysophyta, Haptonema, etc. | Phytoplankton | Phytoplankton | Phytoplankton | Phytoplankton | Phytoplankton | Phytoplankton | Phytoplankton |
| 36 | Detritus | Dissolved Organic Carbon, Particulate Organic Carbon | Detritus | Detritus | Detritus | Detritus | Detritus | Detritus | Detritus |

**Table S2.** **Input data sources and references for FRE’ marine pasture food-web model.** '-' indicates no data.

| Groups No. and name | Bi (t km^-2^) | Pi/Bi (yr^-1^) | Qi/Bi (yr^-1^) | EE | DC |
| --- | --- | --- | --- | --- | --- |
| 1.Marine mammals | (Duan et al., 2009a; Sun et al., 2016) | (Sun et al., 2016) | (Sun et al., 2016) |  | (Sun et al., 2016) |
| 2.Chondrichthyes | (Duan et al., 2009b; Sun et al., 2016) | (Sun et al., 2016) | (Sun et al., 2016) |  | (Sun et al., 2016) |
| 3.Fish-eating birds | (Sun et al., 2016; Lee and Zhang, 2018) | (Sun et al., 2016) | (Sun et al., 2016) |  | (Sun et al., 2016) |
| 4.Flatfishes | Estimated by own group | (Lee and Zhang, 2018) | (Lee and Zhang, 2018) |  | (Lee and Zhang, 2018) |
| 5.Saurida tumbil | Estimated by own group | (Duan et al., 2009a) | (Duan et al., 2009a) |  | (Duan et al., 2009a) |
| 6.Trachurus japonicus | Estimated by own group | (Duan et al., 2009a) | (Duan et al., 2009a) |  | (Duan et al., 2009a) |
| 7.Argyrosomus argentatus | Estimated by own group | (Duan et al., 2009a) | (Duan et al., 2009a) |  | (Duan et al., 2009a) |
| 8.Psenopsis anomala | Estimated by own group | (Duan et al., 2009a) | (Duan et al., 2009a) |  | (Duan et al., 2009a) |
| 9.Thryssa kammalensis | Estimated by own group | (Rahman et al., 2019) | (Rahman et al., 2019) |  | (Rahman et al., 2019) |
| 10.Anglerfish | Estimated by own group | (Rahman et al., 2019) | (Rahman et al., 2019) |  | (Rahman et al., 2019) |
| 11.Other gobiidae | Estimated by own group | (Rahman et al., 2019) | (Rahman et al., 2019) |  | (Rahman et al., 2019) |
| 12.Secutor ruconius | Estimated by own group | (Sun et al., 2016) | (Sun et al., 2016) |  | (Sun et al., 2016) |
| 13.Other Cynoglossidae | Estimated by own group | (Chen et al., 2015) | (Chen et al., 2015) |  | (Chen et al., 2015) |
| 14.Other Trichiurus | Estimated by own group | (Sun et al., 2016) | (Sun et al., 2016) |  | (Sun et al., 2016) |
| 15.Scorpaenidae | Estimated by own group | (Chen et al., 2015) | (Chen et al., 2015) |  | (Chen et al., 2015) |
| 16.Other Synodidae | Estimated by own group | (Chen et al., 2015) | (Chen et al., 2015) |  | (Chen et al., 2015) |
| 17.Tetraodontidae | Estimated by own group | (Chen et al., 2015) | (Chen et al., 2015) |  | (Chen et al., 2015) |
| 18.Other Sciaenidae | Estimated by own group | (Chen et al., 2015) | (Chen et al., 2015) |  | (Chen et al., 2015) |
| 19.Mugilidae | Estimated by own group | (Chen et al., 2015) | (Chen et al., 2015) |  | (Chen et al., 2015) |
| 20.Clupeidae | Estimated by own group | (Chen et al., 2015) | (Chen et al., 2015) |  | (Chen et al., 2015) |
| 21.Engraulidae | Estimated by own group | (Chen et al., 2015) | (Chen et al., 2015) |  | (Chen et al., 2015) |
| 22.Anguilliformes | Estimated by own group | (Lee and Zhang, 2018) | (Lee and Zhang, 2018) |  | (Lee and Zhang, 2018) |
| 23.Other Piscivorous fishes | Estimated by own group | (Sun et al., 2016) | (Sun et al., 2016) |  | (Sun et al., 2016) |
| 24.Other omnivorous fishes | Estimated by own group | (Sun et al., 2016) | (Sun et al., 2016) |  | (Sun et al., 2016) |
| 25.Other demersal fish | Estimated by own group | (Duan et al., 2009a) | (Duan et al., 2009a) |  | (Duan et al., 2009a) |
| 26.Other benthic fishes 1 | Estimated by own group | (Rahman et al., 2019) | (Rahman et al., 2019) |  | (Rahman et al., 2019) |
| 27.Other benthic fishes 2 | Estimated by own group | (Lee and Zhang, 2018) | (Lee and Zhang, 2018) |  | (Lee and Zhang, 2018) |
| 28.Cephalopods | Estimated by own group | (Chen et al., 2015) | (Chen et al., 2015) |  | (Chen et al., 2015) |
| 29.Mantis shrimp | Estimated by own group | (Rahman et al., 2019) | (Rahman et al., 2019) |  | (Rahman et al., 2019) |
| 30.Shrimps | Estimated by own group | (Chen et al., 2015) | (Chen et al., 2015) |  | (Chen et al., 2015) |
| 31.Crabs | Estimated by own group | (Chen et al., 2015) | (Chen et al., 2015) |  | (Chen et al., 2015) |
| 32. Other mollusks | Estimated by own group | (Chen et al., 2015) | (Chen et al., 2015) |  | (Chen et al., 2015) |
| 33.Other Zooplankton | Estimated by own group | (Sun et al., 2016) | (Sun et al., 2016) |  | (Sun et al., 2016) |
| 34.Gastropoda | Estimated by own group | (Lee and Zhang, 2018) | (Lee and Zhang, 2018) |  | (Lee and Zhang, 2018) |
| 35.Phytoplankton | Estimated by own group | (Chen et al., 2015) | - |  | - |
| 36.Detritus | Estimated by own group | - | - |  | - |

**Table S3.** **Parameter input values for each functional group.** TL, trophic level; B, biomass (t km^−2^); P/B, production-to-biomass ratio (year^−1^); Q/B, consumption-to-biomass ratio (year^−1^); EE, ecotrophic efficiency; P/Q, production-to-consumption ratio (year^−1^); Flowtd, flow to detritus ( t km^−2^ year^−1^); NE, net efficiency (ratio between production and assimilated food); PPR(PP), primary production from primary producers required to sustain the group’s production (t km^−2^ year^−1^); PPR(Det), primary production from detritus required to sustain the fisheries (t km^−2^ year^−1^); OI, omnivory index. “-” means no data available

| Group name | TL | B | P/B | Q/B | EE | P/Q | Flowtd | NE | PPR(PP) | PPR(Det) | OI |
| --- | --- | --- | --- | --- | --- | --- | --- | --- | --- | --- | --- |
| **AWAR-BFM** |  |  |  |  |  |  |  |  |  |  |  |
| 1.Mar. mammals | 4.691 | 0.009 | 0.060 | 14.500 | 0.000 | 0.004 | 0.027 | 0.005 | 14.570 | 10.690 | 0.121 |
| 2.Chondrichthyes | 4.356 | 0.040 | 1.120 | 3.840 | 0.291 | 0.292 | 0.062 | 0.365 | 9.357 | 6.872 | 0.214 |
| 3.Fish-eating birds | 4.195 | 0.003 | 0.060 | 61.280 | 0.000 | 0.001 | 0.037 | 0.001 | 5.903 | 4.246 | 0.199 |
| 4.Flatfishes | 3.609 | 0.021 | 1.600 | 3.200 | 0.000 | 0.500 | 0.047 | 0.625 | 7.341 | 5.367 | 0.097 |
| 7.Argyrosomus argentatus | 3.658 | 0.007 | 2.010 | 7.710 | 0.014 | 0.261 | 0.024 | 0.326 | 1.048 | 0.750 | 0.079 |
| 11.Other gobiidae | 3.375 | 0.056 | 1.592 | 4.700 | 0.928 | 0.339 | 0.059 | 0.423 | 2.544 | 1.863 | 0.489 |
| 13.Other Cynoglossidae | 3.314 | 0.042 | 1.604 | 6.975 | 0.003 | 0.230 | 0.124 | 0.287 | 3.131 | 2.260 | 0.343 |
| 15.Scorpaenidae | 4.036 | 0.018 | 1.390 | 5.450 | 0.008 | 0.255 | 0.045 | 0.319 | 2.499 | 1.893 | 0.333 |
| 18.Other Sciaenidae | 3.635 | 0.009 | 1.850 | 10.400 | 0.969 | 0.178 | 0.020 | 0.222 | 1.429 | 1.019 | 0.108 |
| 22.Anguilliformes | 4.148 | 0.003 | 0.568 | 1.315 | 0.140 | 0.432 | 0.002 | 0.540 | 0.072 | 0.052 | 0.238 |
| 23.Other Piscivorous fishes | 4.065 | 0.055 | 1.100 | 5.980 | 0.916 | 0.184 | 0.070 | 0.230 | 8.661 | 6.373 | 0.573 |
| 24.Other omnivorous fishes | 2.185 | 0.096 | 2.980 | 14.200 | 0.158 | 0.210 | 0.515 | 0.262 | 1.502 | 1.308 | 0.258 |
| 26.Other benthic fishes 1 | 3.769 | 0.336 | 1.450 | 5.000 | 0.968 | 0.290 | 0.352 | 0.363 | 18.780 | 13.950 | 0.841 |
| 27.Other benthic fishes 2 | 3.391 | 0.385 | 1.800 | 3.600 | 0.975 | 0.500 | 0.294 | 0.625 | 8.447 | 5.911 | 0.132 |
| 28.Cephalopods | 3.604 | 0.099 | 3.100 | 12.800 | 0.959 | 0.242 | 0.266 | 0.303 | 16.670 | 11.960 | 0.282 |
| 29.Mantis shrimp | 2.992 | 0.099 | 8.000 | 28.900 | 0.369 | 0.277 | 1.075 | 0.346 | 8.874 | 6.640 | 0.346 |
| 30.Shrimps | 2.929 | 0.291 | 6.500 | 16.352 | 0.944 | 0.398 | 1.057 | 0.497 | 34.330 | 25.920 | 0.663 |
| 31.Crabs | 3.252 | 0.330 | 5.650 | 28.500 | 0.986 | 0.198 | 1.909 | 0.248 | 53.460 | 38.110 | 0.285 |
| 32. Other mollusks | 2.674 | 6.850 | 2.600 | 19.200 | 0.987 | 0.135 | 26.532 | 0.169 | 146.700 | 100.000 | 0.414 |
| 33.Other Zooplankton | 2.220 | 2.346 | 82.640 | 275.460 | 0.974 | 0.300 | 134.256 | 0.375 | 329.600 | 200.300 | 0.220 |
| 34.Gastropoda | 2.688 | 2.333 | 1.830 | 12.000 | 0.963 | 0.153 | 5.756 | 0.191 | 97.320 | 70.050 | 0.740 |
| 35.Phytoplankton | 1.000 | 9.396 | 235.000 | - | 0.168 | - | 1836.565 | - | - | - | - |
| 36.Detritus | 1.000 | 17.558 | - | - | 0.121 | - | - | - | - | - | 0.146 |
| **WAR-BFM** |  |  |  |  |  |  |  |  |  |  |  |
| 1.Mar. mammals | 4.709 | 0.009 | 0.009 | 14.500 | 0.000 | 0.004 | 0.027 | 0.005 | 13.89 | 9.981 | 0.138 |
| 2.Chondrichthyes | 4.393 | 0.040 | 0.040 | 3.840 | 0.291 | 0.292 | 0.062 | 0.365 | 9 | 6.461 | 0.271 |
| 3.Fish-eating birds | 4.197 | 0.003 | 0.003 | 61.280 | 0.000 | 0.001 | 0.037 | 0.001 | 7.763 | 5.52 | 0.202 |
| 4.Flatfishes | 3.612 | 0.003 | 0.003 | 3.200 | 0.000 | 0.500 | 0.007 | 0.625 | 2.17 | 1.55 | 0.093 |
| 5.Saurida tumbil | 4.415 | 0.001 | 0.001 | 7.990 | 0.124 | 0.266 | 0.004 | 0.333 | 1.029 | 0.737 | 0.423 |
| 7.Argyrosomus argentatus | 3.658 | 0.003 | 0.003 | 7.710 | 0.005 | 0.261 | 0.012 | 0.326 | 0.52 | 0.37 | 0.079 |
| 9.Kammal thryssa | 3.211 | 0.001 | 0.001 | 11.650 | 0.115 | 0.070 | 0.002 | 0.088 | 0.0352 | 0.0242 | 0.301 |
| 11.Other gobiidae | 3.375 | 0.051 | 0.051 | 4.700 | 0.926 | 0.339 | 0.054 | 0.423 | 2.347 | 1.704 | 0.489 |
| 13.Other Cynoglossidae | 3.314 | 0.028 | 0.028 | 6.975 | 0.003 | 0.230 | 0.084 | 0.287 | 2.198 | 1.575 | 0.343 |
| 15.Scorpaenidae | 4.280 | 0.001 | 0.001 | 5.450 | 0.029 | 0.255 | 0.002 | 0.319 | 0.737 | 0.529 | 0.242 |
| 18.Other Sciaenidae | 3.635 | 0.020 | 0.020 | 10.400 | 0.404 | 0.178 | 0.065 | 0.222 | 3.266 | 2.312 | 0.108 |
| 23.Other Piscivorous fishes | 4.207 | 0.053 | 0.053 | 5.980 | 0.934 | 0.184 | 0.068 | 0.230 | 8.433 | 6.029 | 0.528 |
| 26.Other benthic fishes 1 | 3.769 | 0.302 | 0.302 | 5.000 | 0.985 | 0.290 | 0.308 | 0.363 | 14.85 | 10.93 | 0.841 |
| 27.Other benthic fishes 2 | 3.391 | 0.335 | 0.335 | 3.600 | 0.983 | 0.500 | 0.252 | 0.625 | 7.566 | 5.257 | 0.132 |
| 28.Cephalopods | 3.604 | 0.082 | 0.082 | 12.800 | 0.993 | 0.242 | 0.211 | 0.303 | 13.99 | 9.954 | 0.282 |
| 29.Mantis shrimp | 2.992 | 0.059 | 0.059 | 28.900 | 0.566 | 0.277 | 0.544 | 0.346 | 5.45 | 4.046 | 0.346 |
| 30.Shrimps | 2.929 | 0.231 | 0.231 | 16.352 | 0.994 | 0.398 | 0.765 | 0.497 | 28.65 | 21.45 | 0.663 |
| 31.Crabs | 3.252 | 0.281 | 0.281 | 28.500 | 0.975 | 0.198 | 1.638 | 0.248 | 47.1 | 33.35 | 0.285 |
| 32. Other mollusks | 2.674 | 5.978 | 5.978 | 19.200 | 0.951 | 0.135 | 23.715 | 0.169 | 131.9 | 89.57 | 0.414 |
| 33.Other Zooplankton | 2.220 | 2.249 | 2.249 | 275.460 | 0.936 | 0.300 | 135.786 | 0.375 | 315.9 | 192 | 0.220 |
| 34.Gastropoda | 2.688 | 1.922 | 1.922 | 12.000 | 0.982 | 0.153 | 4.677 | 0.191 | 84.71 | 60.52 | 0.740 |
| 35.Phytoplankton | 1.000 | 8.106 | 8.106 | - | 0.185 | - | 1553.390 | - | - | - | - |
| 36.Detritus | 1.000 | 10.603 | 10.603 | - | 0.132 | - | - | - | - | - | 0.160 |
| **AWAR-AFM** |  |  |  |  |  |  |  |  |  |  |  |
| 1.Mar. mammals | 4.709 | 0.009 | 0.060 | 14.500 | 0.000 | 0.004 | 0.027 | 0.005 | 15.350 | 11.060 | 0.138 |
| 2.Chondrichthyes | 4.393 | 0.040 | 1.120 | 3.840 | 0.291 | 0.292 | 0.062 | 0.365 | 9.980 | 7.184 | 0.271 |
| 3.Fish-eating birds | 4.197 | 0.003 | 0.060 | 61.280 | 0.000 | 0.001 | 0.037 | 0.001 | 7.445 | 5.319 | 0.202 |
| 4.Flatfishes | 3.611 | 0.020 | 1.600 | 3.200 | 0.000 | 0.500 | 0.044 | 0.625 | 12.480 | 9.014 | 0.097 |
| 7.Argyrosomus argentatus | 3.658 | 0.011 | 2.010 | 7.710 | 0.008 | 0.261 | 0.041 | 0.326 | 2.067 | 1.476 | 0.079 |
| 11.Other gobiidae | 3.375 | 0.088 | 1.592 | 4.700 | 0.996 | 0.339 | 0.083 | 0.423 | 4.643 | 3.379 | 0.489 |
| 13.Other Cynoglossidae | 3.314 | 0.027 | 1.604 | 6.975 | 0.061 | 0.230 | 0.079 | 0.287 | 2.355 | 1.693 | 0.343 |
| 14.Other Trichiurus | 3.966 | 0.001 | 1.200 | 4.000 | 0.067 | 0.300 | 0.002 | 0.375 | 0.158 | 0.113 | 0.501 |
| 15.Scorpaenidae | 4.280 | 0.045 | 1.390 | 5.450 | 0.003 | 0.255 | 0.112 | 0.319 | 8.602 | 6.229 | 0.242 |
| 16.Other Synodidae | 3.716 | 0.009 | 1.450 | 6.220 | 0.019 | 0.233 | 0.023 | 0.291 | 1.350 | 0.997 | 0.324 |
| 18.Other Sciaenidae | 3.635 | 0.015 | 1.850 | 10.400 | 0.629 | 0.178 | 0.042 | 0.222 | 2.672 | 1.900 | 0.108 |
| 23.Other Piscivorous fishes | 4.207 | 0.052 | 1.100 | 5.980 | 0.990 | 0.184 | 0.062 | 0.230 | 9.499 | 6.813 | 0.528 |
| 24.Other omnivorous fishes | 2.185 | 0.013 | 2.980 | 14.200 | 0.614 | 0.210 | 0.054 | 0.262 | 0.211 | 0.183 | 0.258 |
| 25.Other demersal fish | 3.444 | 0.001 | 3.458 | 12.885 | 0.461 | 0.268 | 0.005 | 0.335 | 0.172 | 0.121 | 0.608 |
| 26.Other benthic fishes 1 | 3.769 | 0.617 | 1.450 | 5.000 | 0.998 | 0.290 | 0.619 | 0.363 | 32.900 | 24.230 | 0.841 |
| 27.Other benthic fishes 2 | 3.391 | 0.699 | 1.800 | 3.600 | 0.951 | 0.500 | 0.565 | 0.625 | 17.890 | 12.530 | 0.132 |
| 28.Cephalopods | 3.604 | 0.263 | 3.100 | 12.800 | 0.973 | 0.242 | 0.695 | 0.303 | 49.290 | 35.210 | 0.282 |
| 29.Mantis shrimp | 2.992 | 0.099 | 8.000 | 28.900 | 0.638 | 0.277 | 0.862 | 0.346 | 9.831 | 7.309 | 0.346 |
| 30.Shrimps | 2.929 | 0.922 | 6.500 | 16.352 | 0.917 | 0.398 | 3.510 | 0.497 | 123.700 | 92.450 | 0.663 |
| 31.Crabs | 3.252 | 1.152 | 5.650 | 28.500 | 0.832 | 0.198 | 7.663 | 0.248 | 200.800 | 143.000 | 0.285 |
| 32. Other mollusks | 2.674 | 24.870 | 2.600 | 19.200 | 0.940 | 0.135 | 99.392 | 0.169 | 538.500 | 367.500 | 0.414 |
| 33.Other Zooplankton | 2.220 | 8.324 | 82.640 | 275.460 | 0.968 | 0.300 | 480.478 | 0.375 | 1169.000 | 710.800 | 0.220 |
| 34.Gastropoda | 2.688 | 8.083 | 1.830 | 12.000 | 0.891 | 0.153 | 21.014 | 0.191 | 367.100 | 263.100 | 0.740 |
| 35.Phytoplankton | 1.000 | 6.868 | 235.000 | - | 0.817 | - | 296.074 | - | - | - | - |
| 36.Detritus | 1.000 | 31.249 | - | - | 0.940 | - | 0.000 | - | - | - | 0.426 |
| **WAR-AFM** |  |  |  |  |  |  |  |  |  |  |  |
| 1.Mar. mammals | 4.286 | 0.009 | 0.060 | 14.500 | 0.000 | 0.004 | 0.027 | 0.005 | 5.124 | 3.770 | 0.352 |
| 2.Chondrichthyes | 4.030 | 0.040 | 1.120 | 3.840 | 0.291 | 0.292 | 0.062 | 0.365 | 2.869 | 2.100 | 0.078 |
| 3.Fish-eating birds | 4.009 | 0.003 | 0.060 | 61.280 | 0.000 | 0.001 | 0.037 | 0.001 | 4.781 | 3.469 | 0.280 |
| 4.Flatfishes | 3.599 | 0.001 | 1.600 | 3.200 | 0.000 | 0.500 | 0.002 | 0.625 | 8.985 | 6.520 | 0.081 |
| 7.Argyrosomus argentatus | 3.650 | 0.018 | 2.010 | 7.710 | 0.000 | 0.261 | 0.065 | 0.326 | 3.008 | 2.160 | 0.072 |
| 11.Other gobiidae | 3.368 | 0.010 | 1.592 | 4.700 | 0.987 | 0.339 | 0.009 | 0.423 | 0.457 | 0.335 | 0.474 |
| 12.Secutor ruconius | 2.594 | 0.000 | 3.120 | 14.700 | 0.044 | 0.212 | 0.000 | 0.265 | 0.004 | 0.002 | 0.639 |
| 13.Other Cynoglossidae | 3.313 | 0.008 | 1.604 | 6.975 | 0.181 | 0.230 | 0.022 | 0.287 | 0.682 | 0.493 | 0.342 |
| 15.Scorpaenidae | 4.223 | 0.044 | 1.390 | 5.450 | 0.000 | 0.255 | 0.109 | 0.319 | 5.950 | 4.342 | 0.215 |
| 18.Other Sciaenidae | 3.633 | 0.010 | 1.850 | 10.400 | 0.864 | 0.178 | 0.022 | 0.222 | 1.608 | 1.150 | 0.107 |
| 24.Other omnivorous fishes | 2.184 | 0.254 | 2.980 | 14.200 | 0.945 | 0.210 | 0.763 | 0.262 | 3.723 | 3.250 | 0.255 |
| 25.Other demersal fish | 3.228 | 0.347 | 3.458 | 12.885 | 0.960 | 0.268 | 0.943 | 0.335 | 18.370 | 13.110 | 0.363 |
| 26.Other benthic fishes 1 | 3.707 | 0.467 | 1.450 | 5.000 | 0.993 | 0.290 | 0.472 | 0.363 | 19.330 | 14.340 | 0.762 |
| 29.Mantis shrimp | 2.988 | 0.075 | 8.000 | 28.900 | 0.765 | 0.277 | 0.574 | 0.346 | 6.075 | 4.571 | 0.334 |
| 30.Shrimps | 2.929 | 0.296 | 6.500 | 16.352 | 0.949 | 0.398 | 1.067 | 0.497 | 37.860 | 28.500 | 0.662 |
| 31.Crabs | 3.200 | 0.332 | 5.650 | 28.500 | 0.890 | 0.198 | 2.100 | 0.248 | 47.470 | 33.990 | 0.277 |
| 32. Other mollusks | 2.674 | 6.755 | 2.600 | 19.200 | 0.990 | 0.135 | 26.123 | 0.169 | 145.800 | 99.550 | 0.414 |
| 33.Other Zooplankton | 2.220 | 2.364 | 82.640 | 275.460 | 0.971 | 0.300 | 135.968 | 0.375 | 332.100 | 201.900 | 0.220 |
| 34.Gastropoda | 2.686 | 2.389 | 1.830 | 12.000 | 0.859 | 0.153 | 6.349 | 0.191 | 99.530 | 71.740 | 0.732 |
| 35.Phytoplankton | 1.000 | 1.958 | 235.000 | 0.000 | 0.816 | - | 84.862 | - | - | - | - |
| 36.Detritus | 1.000 | 12.896 | - | - | 0.944 | - | 0.000 | - | - | - | 0.433 |
| **AMAR-AFM** |  |  |  |  |  |  |  |  |  |  |  |
| 1.Mar. mammals | 4.709 | 0.009 | 0.060 | 14.500 | 0.000 | 0.004 | 0.027 | 0.005 | 31.180 | 22.460 | 0.138 |
| 2.Chondrichthyes | 4.393 | 0.040 | 1.120 | 3.840 | 0.291 | 0.292 | 0.062 | 0.365 | 22.160 | 15.950 | 0.271 |
| 3.Fish-eating birds | 4.244 | 0.003 | 0.060 | 61.280 | 0.000 | 0.001 | 0.037 | 0.001 | 12.340 | 8.866 | 0.268 |
| 4.Flatfishes | 3.616 | 0.040 | 1.600 | 3.200 | 0.000 | 0.500 | 0.089 | 0.625 | 8.572 | 6.176 | 0.105 |
| 7.Argyrosomus argentatus | 3.658 | 0.011 | 2.010 | 7.710 | 0.017 | 0.261 | 0.039 | 0.326 | 1.644 | 1.178 | 0.079 |
| 11.Other gobiidae | 3.375 | 0.126 | 1.592 | 4.700 | 0.980 | 0.339 | 0.122 | 0.423 | 5.685 | 4.165 | 0.489 |
| 13.Other Cynoglossidae | 3.314 | 0.015 | 1.604 | 6.975 | 0.086 | 0.230 | 0.044 | 0.287 | 1.133 | 0.818 | 0.343 |
| 14.Other Trichiurus | 4.089 | 0.005 | 1.200 | 4.000 | 0.067 | 0.300 | 0.010 | 0.375 | 0.663 | 0.472 | 0.448 |
| 15.Scorpaenidae | 4.380 | 0.032 | 1.390 | 5.450 | 0.009 | 0.255 | 0.078 | 0.319 | 4.886 | 3.560 | 0.158 |
| 17Tetraodontidae | 3.462 | 0.002 | 1.660 | 9.560 | 0.107 | 0.174 | 0.007 | 0.217 | 0.207 | 0.149 | 0.447 |
| 21.Engraulidae | 2.528 | 0.001 | 2.890 | 12.800 | 0.157 | 0.226 | 0.004 | 0.282 | 0.012 | 0.007 | 0.365 |
| 22.Anguilliformes | 4.148 | 0.014 | 0.568 | 1.315 | 0.081 | 0.432 | 0.011 | 0.540 | 0.361 | 0.262 | 0.238 |
| 23.Other Piscivorous fishes | 4.207 | 0.236 | 1.100 | 5.980 | 0.273 | 0.184 | 0.470 | 0.230 | 34.700 | 24.940 | 0.528 |
| 25.Other demersal fish | 3.444 | 0.005 | 3.458 | 12.885 | 0.409 | 0.268 | 0.025 | 0.335 | 0.700 | 0.490 | 0.608 |
| 26.Other benthic fishes 1 | 3.769 | 0.904 | 1.450 | 5.000 | 0.983 | 0.290 | 0.927 | 0.363 | 38.360 | 28.430 | 0.841 |
| 27.Other benthic fishes 2 | 3.391 | 0.998 | 1.800 | 3.600 | 0.936 | 0.500 | 0.833 | 0.625 | 21.840 | 15.280 | 0.132 |
| 28.Cephalopods | 3.604 | 0.195 | 3.100 | 12.800 | 0.979 | 0.242 | 0.511 | 0.303 | 31.130 | 22.290 | 0.282 |
| 29.Mantis shrimp | 2.992 | 0.096 | 8.000 | 28.900 | 0.967 | 0.277 | 0.579 | 0.346 | 8.488 | 6.355 | 0.346 |
| 30.Shrimps | 2.929 | 0.587 | 6.500 | 16.352 | 0.918 | 0.398 | 2.232 | 0.497 | 68.110 | 51.480 | 0.663 |
| 31.Crabs | 3.252 | 0.701 | 5.650 | 28.500 | 0.957 | 0.198 | 4.168 | 0.248 | 111.000 | 79.130 | 0.285 |
| 32. Other mollusks | 2.674 | 13.219 | 2.600 | 19.200 | 0.980 | 0.135 | 51.449 | 0.169 | 281.900 | 192.200 | 0.414 |
| 33.Other Zooplankton | 2.220 | 4.468 | 82.640 | 275.460 | 0.978 | 0.300 | 254.266 | 0.375 | 627.700 | 381.500 | 0.220 |
| 34.Gastropoda | 2.688 | 4.323 | 1.830 | 12.000 | 0.998 | 0.153 | 10.392 | 0.191 | 182.100 | 131.100 | 0.740 |
| 35.Phytoplankton | 1.000 | 3.756 | 235.000 | - | 0.801 | - | 175.712 | - | - | - | - |
| 36.Detritus | 1.000 | 15.094 | - | - | 0.918 | - | 0.000 | - | - | - | 0.452 |
| **MAR-AFM** |  |  |  |  |  |  |  |  |  |  |  |
| 1.Mar. mammals | 4.709 | 0.009 | 0.060 | 14.500 | 0.000 | 0.004 | 0.027 | 0.005 | 13.610 | 9.816 | 0.138 |
| 2.Chondrichthyes | 4.393 | 0.040 | 1.120 | 3.840 | 0.291 | 0.292 | 0.062 | 0.365 | 8.886 | 6.403 | 0.271 |
| 3.Fish-eating birds | 4.197 | 0.003 | 0.060 | 61.280 | 0.000 | 0.001 | 0.037 | 0.001 | 6.204 | 4.438 | 0.202 |
| 4.Flatfishes | 3.616 | 0.118 | 1.600 | 3.200 | 0.000 | 0.500 | 0.264 | 0.625 | 20.600 | 14.850 | 0.102 |
| 5.Saurida tumbil | 4.415 | 0.003 | 2.126 | 7.990 | 0.289 | 0.266 | 0.010 | 0.333 | 0.709 | 0.511 | 0.423 |
| 6.Trachurus japonicus | 3.883 | 0.002 | 2.150 | 10.471 | 0.265 | 0.205 | 0.007 | 0.257 | 0.593 | 0.428 | 0.432 |
| 7.Argyrosomus argentatus | 3.658 | 0.022 | 2.010 | 7.710 | 0.025 | 0.261 | 0.078 | 0.326 | 3.293 | 2.356 | 0.079 |
| 10.Anglerfish | 4.343 | 0.000 | 1.160 | 3.800 | 0.328 | 0.305 | 0.000 | 0.382 | 0.057 | 0.041 | 0.373 |
| 11.Other gobiidae | 3.375 | 0.093 | 1.592 | 4.700 | 0.920 | 0.339 | 0.099 | 0.423 | 4.008 | 2.931 | 0.489 |
| 13.Other Cynoglossidae | 3.314 | 0.033 | 1.604 | 6.975 | 0.084 | 0.230 | 0.095 | 0.287 | 2.443 | 1.762 | 0.343 |
| 15.Scorpaenidae | 4.280 | 0.059 | 1.390 | 5.450 | 0.014 | 0.255 | 0.144 | 0.319 | 8.666 | 6.297 | 0.242 |
| 16.Other Synodidae | 3.716 | 0.006 | 1.450 | 6.220 | 0.136 | 0.233 | 0.015 | 0.291 | 1.023 | 0.790 | 0.324 |
| 17.Tetraodontidae | 3.447 | 0.003 | 1.660 | 9.560 | 0.000 | 0.174 | 0.010 | 0.217 | 1.736 | 1.257 | 0.419 |
| 18.Other Sciaenidae | 3.635 | 0.015 | 1.850 | 10.400 | 0.644 | 0.178 | 0.041 | 0.222 | 2.232 | 1.588 | 0.108 |
| 19.Mugilidae | 3.030 | 0.039 | 2.550 | 15.500 | 0.017 | 0.165 | 0.216 | 0.206 | 4.535 | 3.293 | 0.642 |
| 21.Engraulidae | 2.528 | 0.003 | 2.890 | 12.800 | 0.609 | 0.226 | 0.013 | 0.282 | 0.050 | 0.027 | 0.365 |
| 23.Other Piscivorous fishes | 4.207 | 0.062 | 1.100 | 5.980 | 0.864 | 0.184 | 0.083 | 0.230 | 9.274 | 6.658 | 0.528 |
| 24.Other omnivorous fishes | 2.185 | 0.026 | 2.980 | 14.200 | 0.227 | 0.210 | 0.134 | 0.262 | 0.377 | 0.331 | 0.258 |
| 25.Other demersal fish | 3.444 | 0.003 | 3.458 | 12.885 | 0.218 | 0.268 | 0.015 | 0.335 | 0.377 | 0.264 | 0.608 |
| 26.Other benthic fishes 1 | 3.769 | 0.594 | 1.450 | 5.000 | 0.961 | 0.290 | 0.628 | 0.363 | 25.080 | 18.550 | 0.841 |
| 27.Other benthic fishes 2 | 3.391 | 0.681 | 1.800 | 3.600 | 0.917 | 0.500 | 0.592 | 0.625 | 14.800 | 10.340 | 0.132 |
| 28.Cephalopods | 3.604 | 0.139 | 3.100 | 12.800 | 0.967 | 0.242 | 0.369 | 0.303 | 22.070 | 15.790 | 0.282 |
| 29.Mantis shrimp | 2.992 | 0.064 | 8.000 | 28.900 | 0.962 | 0.277 | 0.390 | 0.346 | 5.684 | 4.253 | 0.346 |
| 30.Shrimps | 2.929 | 0.404 | 6.500 | 16.352 | 0.995 | 0.398 | 1.333 | 0.497 | 46.760 | 35.360 | 0.663 |
| 31.Crabs | 3.252 | 0.495 | 5.650 | 28.500 | 0.919 | 0.198 | 3.050 | 0.248 | 77.560 | 55.240 | 0.285 |
| 32. Other mollusks | 2.674 | 9.807 | 2.600 | 19.200 | 0.993 | 0.135 | 37.828 | 0.169 | 209.600 | 142.800 | 0.414 |
| 33.Other Zooplankton | 2.220 | 3.318 | 82.640 | 275.460 | 0.976 | 0.300 | 189.312 | 0.375 | 466.100 | 283.300 | 0.220 |
| 34.Gastropoda | 2.688 | 3.280 | 1.830 | 12.000 | 0.997 | 0.153 | 7.889 | 0.191 | 137.200 | 98.640 | 0.740 |
| 35.Phytoplankton | 1.000 | 2.907 | 235.000 | - | 0.769 | - | 157.922 | - | - | - | - |
| 36.Detritus | 1.000 | 29.195 | - | - | 0.855 | - | 0.000 | - | - | - | 0.469 |
| **FAFAR-AFM** |  |  |  |  |  |  |  |  |  |  |  |
| 1.Mar. mammals | 4.709 | 0.009 | 0.060 | 14.500 | 0.000 | 0.004 | 0.027 | 0.005 | 13.730 | 9.920 | 0.138 |
| 2.Chondrichthyes | 4.393 | 0.040 | 1.120 | 3.840 | 0.291 | 0.292 | 0.062 | 0.365 | 8.912 | 6.434 | 0.271 |
| 3.Fish-eating birds | 4.197 | 0.003 | 0.060 | 61.280 | 0.000 | 0.001 | 0.037 | 0.001 | 5.774 | 4.137 | 0.202 |
| 4.Flatfishes | 3.607 | 0.025 | 1.600 | 3.200 | 0.000 | 0.500 | 0.056 | 0.625 | 5.194 | 3.730 | 0.088 |
| 7.Argyrosomus argentatus | 3.658 | 0.002 | 2.010 | 7.710 | 0.075 | 0.261 | 0.005 | 0.326 | 0.247 | 0.177 | 0.079 |
| 8.Psenopsis anomala | 2.244 | 0.001 | 1.400 | 27.270 | 0.139 | 0.051 | 0.008 | 0.064 | 0.039 | 0.007 | 0.238 |
| 11.Other gobiidae | 3.375 | 0.068 | 1.592 | 4.700 | 0.936 | 0.339 | 0.071 | 0.423 | 3.063 | 2.239 | 0.489 |
| 13.Other Cynoglossidae | 3.314 | 0.005 | 1.604 | 6.975 | 0.256 | 0.230 | 0.013 | 0.287 | 0.389 | 0.280 | 0.343 |
| 15.Scorpaenidae | 4.280 | 0.031 | 1.390 | 5.450 | 0.006 | 0.255 | 0.076 | 0.319 | 4.151 | 3.024 | 0.242 |
| 17.Tetraodontidae | 3.462 | 0.001 | 1.660 | 9.560 | 0.000 | 0.174 | 0.004 | 0.217 | 0.113 | 0.081 | 0.447 |
| 18.Other Sciaenidae | 3.635 | 0.009 | 1.850 | 10.400 | 0.956 | 0.178 | 0.019 | 0.222 | 1.379 | 0.982 | 0.108 |
| 20.Clupeidae | 3.037 | 0.000 | 2.650 | 16.500 | 0.191 | 0.161 | 0.003 | 0.201 | 0.013 | 0.007 | 0.190 |
| 23.Other Piscivorous fishes | 4.207 | 0.054 | 1.100 | 5.980 | 0.920 | 0.184 | 0.070 | 0.230 | 8.474 | 6.093 | 0.528 |
| 24.Other omnivorous fishes | 2.185 | 0.012 | 2.980 | 14.200 | 0.000 | 0.210 | 0.068 | 0.262 | 0.173 | 0.152 | 0.258 |
| 25.Other demersal fish | 3.444 | 0.004 | 3.458 | 12.885 | 0.205 | 0.268 | 0.019 | 0.335 | 0.492 | 0.345 | 0.608 |
| 26.Other benthic fishes 1 | 3.769 | 0.429 | 1.450 | 5.000 | 0.963 | 0.290 | 0.452 | 0.363 | 19.730 | 14.620 | 0.841 |
| 27.Other benthic fishes 2 | 3.391 | 0.466 | 1.800 | 3.600 | 0.959 | 0.500 | 0.369 | 0.625 | 10.330 | 7.223 | 0.132 |
| 28.Cephalopods | 3.604 | 0.102 | 3.100 | 12.800 | 0.982 | 0.242 | 0.267 | 0.303 | 16.990 | 12.160 | 0.282 |
| 29.Mantis shrimp | 2.992 | 0.067 | 8.000 | 28.900 | 0.681 | 0.277 | 0.557 | 0.346 | 6.015 | 4.501 | 0.346 |
| 30.Shrimps | 2.929 | 0.292 | 6.500 | 16.352 | 0.984 | 0.398 | 0.985 | 0.497 | 35.300 | 26.620 | 0.663 |
| 31.Crabs | 3.252 | 0.352 | 5.650 | 28.500 | 0.937 | 0.198 | 2.131 | 0.248 | 57.080 | 40.670 | 0.285 |
| 32. Other mollusks | 2.674 | 7.396 | 2.600 | 19.200 | 0.947 | 0.135 | 29.419 | 0.169 | 156.400 | 106.700 | 0.414 |
| 33.Other Zooplankton | 2.220 | 2.395 | 82.640 | 275.460 | 0.991 | 0.300 | 133.790 | 0.375 | 336.500 | 204.500 | 0.220 |
| 34.Gastropoda | 2.688 | 2.342 | 1.830 | 12.000 | 0.973 | 0.153 | 5.736 | 0.191 | 100.400 | 72.120 | 0.740 |
| 35.Phytoplankton | 1.000 | 1.944 | 235.000 | - | 0.833 | - | 76.176 | - | - | - | - |
| 36.Detritus | 1.000 | 20.722 | - | - | 0.992 | - | - | - | - | - | 0.428 |

**Table S4. Diet composition matrix** **for all function groups of 7 different spatio-temporal models in the PRE marine pasture in 2020.** '-' indicates No predator-prey relationship.

| Prey groups | Predator groups | | | | | | | | | | | | | | | | | | | | | | | | | | | |
| --- | --- | --- | --- | --- | --- | --- | --- | --- | --- | --- | --- | --- | --- | --- | --- | --- | --- | --- | --- | --- | --- | --- | --- | --- | --- | --- | --- | --- |
| **AWAR-BFM** | 1 | 2 | 3 | 4 | 7 | 11 | 13 | 15 | 18 | 22 | 23 | 24 | 26 | 27 | 28 | 29 | 30 | 31 | 32 | 33 | 34 |  |  |  |  |  |  |  |
| 1 | - | - | - | - | - | - | - | - | - | - | - | - | - | - | - | - | - | - | - | - | - |  |  |  |  |  |  |  |
| 2 | 0.10 | - | - | - | - | - | - | - | - | - | - | - | - | - | - | - | - | - | - | - | - |  |  |  |  |  |  |  |
| 3 | - | - | - | - | - | - | - | - | - | - | - | - | - | - | - | - | - | - | - | - | - |  |  |  |  |  |  |  |
| 4 | - | - | - | - | - | - | - | - | - | - | - | - | - | - | - | - | - | - | - | - | - |  |  |  |  |  |  |  |
| 7 | - | - | - | 0.00 | - | - | - | - | - | - | - | - | - | - | - | - | - | - | - | - | - |  |  |  |  |  |  |  |
| 11 | - | - | 0.08 | 0.00 | - | - | - | - | - | - | - | - | 0.04 | - | - | - | - | - | - | - | - |  |  |  |  |  |  |  |
| 13 | - | - | - | 0.00 | - | - | - | - | - | - | - | - | - | - | - | - | - | - | - | - | - |  |  |  |  |  |  |  |
| 15 | - | - | - | 0.00 | - | - | - | - | - | - | - | - | - | - | - | - | - | - | - | - | - |  |  |  |  |  |  |  |
| 18 | - | - | 0.08 | 0.00 | - | - | - | 0.01 | - | - | - | - | - | - | - | - | - | - | - | - | - |  |  |  |  |  |  |  |
| 22 | - | - | - | 0.00 | - | - | - | - | - | - | - | - | - | - | - | - | - | - | - | - | - |  |  |  |  |  |  |  |
| 23 | 0.10 | 0.26 | 0.01 | 0.01 | - | - | - | - | - | - | - | - | - | - | - | - | - | - | - | - | - |  |  |  |  |  |  |  |
| 24 | - | - | - | 0.01 | - | - | - | 0.16 | - | - | 0.09 | - | - | - | - | - | - | - | - | - | - |  |  |  |  |  |  |  |
| 26 | 0.38 | 0.01 | 0.04 | 0.01 | - | 0.02 | - | 0.24 | 0.04 | 0.21 | 0.17 | 0.01 | 0.14 | - | 0.07 | 0.00 | - | - | - | - | - |  |  |  |  |  |  |  |
| 27 | 0.29 | 0.02 | 0.12 | 0.01 | - | 0.02 | - | 0.17 | 0.04 | 0.21 | 0.15 | 0.01 | 0.26 | 0.00 | 0.06 | 0.00 | - | - | - | - | - |  |  |  |  |  |  |  |
| 28 | 0.08 | 0.05 | 0.12 | 0.01 | - | - | - | - | - | - | 0.01 | - | 0.01 | 0.01 | - | 0.01 | - | 0.02 | - | - | - |  |  |  |  |  |  |  |
| 29 | 0.01 | 0.17 | 0.05 | 0.01 | - | - | - | - | - | - | - | - | 0.15 | - | - | - | - | - | - | - | - |  |  |  |  |  |  |  |
| 30 | 0.01 | 0.22 | 0.05 | 0.01 | - | 0.30 | - | 0.07 | - | 0.20 | - | - | 0.08 | 0.08 | - | 0.00 | - | - | - | - | 0.05 |  |  |  |  |  |  |  |
| 31 | 0.01 | 0.24 | 0.27 | 0.01 | 0.11 | 0.08 | - | 0.08 | - | 0.18 | 0.49 | 0.00 | - | 0.09 | - | 0.01 | - | - | - | - | 0.05 |  |  |  |  |  |  |  |
| 32 | 0.02 | 0.03 | 0.06 | 0.67 | 0.02 | - | 0.25 | 0.10 | 0.24 | 0.00 | 0.00 | 0.00 | - | 0.01 | 0.24 | 0.03 | 0.29 | 0.22 | 0.05 | - | 0.25 |  |  |  |  |  |  |  |
| 33 | - | - | 0.12 | 0.25 | 0.20 | 0.44 | 0.25 | 0.05 | 0.24 | 0.14 | - | 0.11 | 0.15 | 0.81 | 0.25 | 0.68 | 0.06 | 0.53 | 0.47 | 0.18 | 0.05 |  |  |  |  |  |  |  |
| 34 | - | - | - | 0.01 | 0.67 | - | 0.35 | 0.12 | 0.46 | 0.06 | - | - | - | - | 0.35 | 0.03 | 0.22 | 0.11 | 0.01 | - | - |  |  |  |  |  |  |  |
| 35 | - | - | - | - | - | 0.08 | - | - | - | - | 0.08 | 0.40 | - | - | - | - | - | - | 0.25 | 0.51 | 0.30 |  |  |  |  |  |  |  |
| 36 | - | - | - | - | - | 0.07 | 0.15 | - | - | - | 0.01 | 0.47 | 0.17 | - | 0.05 | 0.23 | 0.43 | 0.12 | 0.22 | 0.31 | 0.30 |  |  |  |  |  |  |  |
| sum | 1 | 1 | 1 | 1 | 1 | 1 | 1 | 1 | 1 | 1 | 1 | 1 | 1 | 1 | 1 | 1 | 1 | 1 | 1 | 1 | 1 |  |  |  |  |  |  |  |
| **WAR-BFM** | 1 | 2 | 3 | 4 | 5 | 7 | 9 | 11 | 13 | 15 | 18 | 23 | 26 | 27 | 28 | 29 | 30 | 31 | 32 | 33 | 34 |  |  |  |  |  |  |  |
| 1 | - | - | - | - | - | - | - | - | - | - | - | - | - | - | - | - | - | - | - | - | - |  |  |  |  |  |  |  |
| 2 | 0.10 | - | - | - | - | - | - | - | - | - | - | - | - | - | - | - | - | - | - | - | - |  |  |  |  |  |  |  |
| 3 | - | - | - | - | - | - | - | - | - | - | - | - | - | - | - | - | - | - | - | - | - |  |  |  |  |  |  |  |
| 4 | - | - | - | - | - | - | - | - | - | - | - | - | - | - | - | - | - | - | - | - | - |  |  |  |  |  |  |  |
| 5 | - | - | - | - | 0.03 | - | - | - | - | - | - | - | - | - | - | - | - | - | - | - | - |  |  |  |  |  |  |  |
| 7 | - | - | - | 0.00 | - | - | - | - | - | - | - | - | - | - | - | - | - | - | - | - | - |  |  |  |  |  |  |  |
| 9 | - | - | - | 0.01 | - | - | - | - | - | - | - | - | - | - | - | - | - | - | - | - | - |  |  |  |  |  |  |  |
| 11 | - | - | 0.08 | 0.01 | 0.01 | - | - | - | - | - | - | - | 0.04 | - | - | - | - | - | - | - | - |  |  |  |  |  |  |  |
| 13 | - | - | - | 0.01 | 0.01 | - | - | - | - | 0.01 | - | - | - | - | - | - | - | - | - | - | - |  |  |  |  |  |  |  |
| 15 | - | - | - | 0.00 | - | - | - | - | - | - | - | - | - | - | - | - | - | - | - | - | - |  |  |  |  |  |  |  |
| 18 | - | - | 0.08 | 0.00 | - | - | - | - | - | - | - | - | - | - | - | - | - | - | - | - | - |  |  |  |  |  |  |  |
| 23 | 0.10 | 0.26 | 0.01 | 0.00 | 0.01 | - | - | - | - | - | - | - | - | - | - | - | - | - | - | - | - |  |  |  |  |  |  |  |
| 26 | 0.38 | 0.01 | 0.04 | 0.01 | 0.23 | - | - | 0.02 | - | 0.40 | 0.04 | 0.26 | 0.14 | - | 0.07 | 0.00 | - | - | - | - | - |  |  |  |  |  |  |  |
| 27 | 0.29 | 0.02 | 0.12 | 0.01 | 0.46 | - | - | 0.02 | - | 0.17 | 0.04 | 0.15 | 0.26 | 0.00 | 0.06 | 0.00 | - | - | - | - | - |  |  |  |  |  |  |  |
| 28 | 0.08 | 0.05 | 0.12 | 0.01 | 0.20 | - | - | - | - | - | - | 0.01 | 0.01 | 0.01 | - | 0.01 | - | 0.02 | - | - | - |  |  |  |  |  |  |  |
| 29 | 0.01 | 0.17 | 0.05 | 0.01 | - | - | 0.04 | - | - | - | - | - | 0.15 | - | - | - | - | - | - | - | - |  |  |  |  |  |  |  |
| 30 | 0.01 | 0.22 | 0.05 | 0.01 | - | - | 0.05 | 0.30 | - | 0.07 | - | - | 0.08 | 0.08 | - | 0.00 | - | - | - | - | 0.05 |  |  |  |  |  |  |  |
| 31 | 0.01 | 0.24 | 0.27 | 0.01 | - | 0.11 | 0.04 | 0.08 | - | 0.08 | - | 0.49 | - | 0.09 | - | 0.01 | - | - | - | - | 0.05 |  |  |  |  |  |  |  |
| 32 | 0.02 | 0.03 | 0.06 | 0.67 | - | 0.02 | 0.04 | - | 0.25 | 0.10 | 0.24 | 0.00 | - | 0.01 | 0.24 | 0.03 | 0.29 | 0.22 | 0.05 | - | 0.25 |  |  |  |  |  |  |  |
| 33 | - | - | 0.12 | 0.25 | - | 0.20 | 0.66 | 0.44 | 0.25 | 0.05 | 0.24 | - | 0.15 | 0.81 | 0.25 | 0.68 | 0.06 | 0.53 | 0.47 | 0.18 | 0.05 |  |  |  |  |  |  |  |
| 34 | - | - | - | 0.01 | - | 0.67 | 0.04 | - | 0.35 | 0.12 | 0.46 | - | - | - | 0.35 | 0.03 | 0.22 | 0.11 | 0.01 | - | - |  |  |  |  |  |  |  |
| 35 | - | - | - | - | 0.01 | - | 0.11 | 0.08 | - | - | - | 0.08 | - | - | - | - | - | - | 0.25 | 0.51 | 0.30 |  |  |  |  |  |  |  |
| 36 | - | - | - | - | 0.05 | - | 0.02 | 0.07 | 0.15 | - | - | 0.01 | 0.17 | - | 0.05 | 0.23 | 0.43 | 0.12 | 0.22 | 0.31 | 0.30 |  |  |  |  |  |  |  |
| sum | 1 | 1 | 1 | 1 | 1 | 1 | 1 | 1 | 1 | 1 | 1 | 1 | 1 | 1 | 1 | 1 | 1 | 1 | 1 | 1 | 1 |  |  |  |  |  |  |  |
| **AWAR-AFM** | 1 | 2 | 3 | 4 | 7 | 11 | 13 | 14 | 15 | 16 | 18 | 23 | 24 | 25 | 26 | 27 | 28 | 29 | 30 | 31 | 32 | 33 | 34 |  |  |  |  |  |
| 1 | - | - | - | - | - | - | - | - | - | - | - | - | - | - | - | - | - | - | - | - | - | - | - |  |  |  |  |  |
| 2 | 0.10 | - | - | - | - | - | - | - | - | - | - | - | - | - | - | - | - | - | - | - | - | - | - |  |  |  |  |  |
| 3 | - | - | - | - | - | - | - | - | - | - | - | - | - | - | - | - | - | - | - | - | - | - | - |  |  |  |  |  |
| 4 | - | - | - | - | - | - | - | - | - | - | - | - | - | - | - | - | - | - | - | - | - | - | - |  |  |  |  |  |
| 7 | - | - | - | 0.00 | - | - | - | - | - | - | - | - | - | - | - | - | - | - | - | - | - | - | - |  |  |  |  |  |
| 11 | - | - | 0.08 | 0.00 | - | - | - | - | - | - | - | - | - | - | 0.04 | - | - | - | - | - | - | - | - |  |  |  |  |  |
| 13 | - | - | - | 0.00 | - | - | - | - | 0.01 | - | - | - | - | - | - | - | - | - | - | - | - | - | - |  |  |  |  |  |
| 14 | - | - | - | - | - | - | - | 0.02 | - | - | - | - | - | - | - | - | - | - | - | - | - | - | - |  |  |  |  |  |
| 15 | - | - | - | 0.00 | - | - | - | - | - | - | - | - | - | - | - | - | - | - | - | - | - | - | - |  |  |  |  |  |
| 16 | - | - | - | 0.00 | - | - | - | - | - | 0.00 | - | - | - | - | - | - | - | - | - | - | - | - | - |  |  |  |  |  |
| 18 | - | - | 0.08 | 0.00 | - | - | - | - | - | 0.04 | - | - | - | - | - | - | - | - | - | - | - | - | - |  |  |  |  |  |
| 23 | 0.10 | 0.26 | 0.01 | 0.00 | - | - | - | - | - | 0.03 | - | - | - | - | - | - | - | - | - | - | - | - | - |  |  |  |  |  |
| 24 | - | - | - | 0.00 | - | - | - | 0.10 | - | 0.45 | - | - | - | - | - | - | - | - | - | - | - | - | - |  |  |  |  |  |
| 25 | - | - | - | 0.00 | - | - | - | 0.18 | - | - | - | - | - | 0.06 | - | - | - | - | - | - | - | - | - |  |  |  |  |  |
| 26 | 0.38 | 0.01 | 0.04 | 0.01 | - | 0.02 | - | 0.06 | 0.40 | 0.03 | 0.04 | 0.26 | 0.01 | 0.03 | 0.14 | - | 0.07 | 0.00 | - | - | - | - | - |  |  |  |  |  |
| 27 | 0.29 | 0.02 | 0.12 | 0.01 | - | 0.02 | - | 0.06 | 0.17 | 0.06 | 0.04 | 0.15 | 0.01 | 0.04 | 0.26 | 0.00 | 0.06 | 0.00 | - | - | - | - | - |  |  |  |  |  |
| 28 | 0.08 | 0.05 | 0.12 | 0.01 | - | - | - | 0.01 | - | 0.02 | - | 0.01 | - | 0.11 | 0.01 | 0.01 | - | 0.01 | - | 0.02 | - | - | - |  |  |  |  |  |
| 29 | 0.01 | 0.17 | 0.05 | 0.01 | - | - | - | 0.10 | - | - | - | - | - | 0.02 | 0.15 | - | - | - | - | - | - | - | - |  |  |  |  |  |
| 30 | 0.01 | 0.22 | 0.05 | 0.01 | - | 0.30 | - | 0.20 | 0.07 | 0.11 | - | - | - | 0.03 | 0.08 | 0.08 | - | 0.00 | - | - | - | - | 0.05 |  |  |  |  |  |
| 31 | 0.01 | 0.24 | 0.27 | 0.01 | 0.11 | 0.08 | - | 0.19 | 0.08 | 0.12 | - | 0.49 | 0.00 | 0.02 | - | 0.09 | - | 0.01 | - | - | - | - | 0.05 |  |  |  |  |  |
| 32 | 0.02 | 0.03 | 0.06 | 0.67 | 0.02 | - | 0.25 | - | 0.10 | 0.16 | 0.24 | 0.00 | 0.00 | 0.02 | - | 0.01 | 0.24 | 0.03 | 0.29 | 0.22 | 0.05 | - | 0.25 |  |  |  |  |  |
| 33 | - | - | 0.12 | 0.25 | 0.20 | 0.44 | 0.25 | - | 0.05 | - | 0.24 | - | 0.11 | 0.53 | 0.15 | 0.81 | 0.25 | 0.68 | 0.06 | 0.53 | 0.47 | 0.18 | 0.05 |  |  |  |  |  |
| 34 | - | - | - | 0.01 | 0.67 | - | 0.35 | - | 0.12 | - | 0.46 | - | - | 0.02 | - | - | 0.35 | 0.03 | 0.22 | 0.11 | 0.01 | - | - |  |  |  |  |  |
| 35 | - | - | - | - | - | 0.08 | - | 0.08 | - | - | - | 0.08 | 0.40 | 0.13 | - | - | - | - | - | - | 0.25 | 0.51 | 0.30 |  |  |  |  |  |
| 36 | - | - | - | - | - | 0.07 | 0.15 | - | - | - | - | 0.01 | 0.47 | - | 0.17 | - | 0.05 | 0.23 | 0.43 | 0.12 | 0.22 | 0.31 | 0.30 |  |  |  |  |  |
| sum | 1 | 1 | 1 | 1 | 1 | 1 | 1 | 1 | 1 | 1 | 1 | 1 | 1 | 1 | 1 | 1 | 1 | 1 | 1 | 1 | 1 | 1 | 1 |  |  |  |  |  |
| **WAR-AFM** | 1 | 2 | 3 | 4 | 7 | 11 | 12 | 13 | 15 | 18 | 24 | 25 | 26 | 29 | 30 | 31 | 32 | 33 | 34 |  |  |  |  |  |  |  |  |  |
| 1 | - | - | - | - | - | - | - | - | - | - | - | - | - | - | - | - | - | - | - |  |  |  |  |  |  |  |  |  |
| 2 | 0.10 | - | - | - | - | - | - | - | - | - | - | - | - | - | - | - | - | - | - |  |  |  |  |  |  |  |  |  |
| 3 | - | - | - | - | - | - | - | - | - | - | - | - | - | - | - | - | - | - | - |  |  |  |  |  |  |  |  |  |
| 4 | - | - | - | - | - | - | - | - | - | - | - | - | - | - | - | - | - | - | - |  |  |  |  |  |  |  |  |  |
| 7 | - | - | - | 0.01 | - | - | - | - | - | - | - | - | - | - | - | - | - | - | - |  |  |  |  |  |  |  |  |  |
| 11 | - | - | 0.08 | 0.00 | - | - | - | - | - | - | - | - | - | - | - | - | - | - | - |  |  |  |  |  |  |  |  |  |
| 12 | - | - | - | 0.00 | - | - | - | - | - | - | - | - | - | - | - | - | - | - | - |  |  |  |  |  |  |  |  |  |
| 13 | - | - | - | 0.00 | - | - | - | - | 0.01 | - | - | - | - | - | - | - | - | - | - |  |  |  |  |  |  |  |  |  |
| 15 | - | - | - | 0.01 | - | - | - | - | - | - | - | - | - | - | - | - | - | - | - |  |  |  |  |  |  |  |  |  |
| 18 | - | - | 0.08 | 0.00 | - | - | - | - | - | - | - | - | - | - | - | - | - | - | - |  |  |  |  |  |  |  |  |  |
| 24 | 0.18 | 0.07 | 0.13 | 0.01 | - | - | - | - | - | - | - | 0.15 | - | - | - | - | - | - | - |  |  |  |  |  |  |  |  |  |
| 25 | 0.29 | 0.26 | 0.04 | 0.01 | - | 0.02 | - | - | 0.17 | 0.02 | 0.01 | 0.06 | 0.31 | 0.01 | - | - | - | - | - |  |  |  |  |  |  |  |  |  |
| 26 | 0.38 | 0.01 | 0.12 | 0.00 | - | 0.02 | - | - | 0.40 | 0.05 | 0.01 | 0.03 | 0.14 | 0.00 | - | - | - | - | - |  |  |  |  |  |  |  |  |  |
| 29 | 0.01 | 0.17 | 0.05 | 0.01 | - | - | 0.06 | - | - | - | - | 0.02 | 0.15 | - | - | - | - | - | - |  |  |  |  |  |  |  |  |  |
| 30 | 0.01 | 0.22 | 0.05 | 0.01 | - | 0.30 | 0.05 | - | 0.07 | - | - | 0.03 | 0.08 | 0.00 | - | - | - | - | 0.05 |  |  |  |  |  |  |  |  |  |
| 31 | 0.01 | 0.24 | 0.27 | 0.01 | 0.11 | 0.08 | 0.05 | - | 0.08 | - | 0.00 | 0.02 | - | 0.01 | - | - | - | - | 0.05 |  |  |  |  |  |  |  |  |  |
| 32 | 0.02 | 0.03 | 0.06 | 0.67 | 0.02 | - | - | 0.25 | 0.10 | 0.24 | 0.00 | 0.02 | - | 0.03 | 0.29 | 0.22 | 0.05 | - | 0.25 |  |  |  |  |  |  |  |  |  |
| 33 | - | - | 0.12 | 0.25 | 0.20 | 0.44 | 0.22 | 0.25 | 0.05 | 0.24 | 0.11 | 0.53 | 0.15 | 0.68 | 0.06 | 0.53 | 0.47 | 0.18 | 0.05 |  |  |  |  |  |  |  |  |  |
| 34 | - | - | - | 0.01 | 0.67 | - | - | 0.35 | 0.12 | 0.46 | - | 0.02 | - | 0.03 | 0.22 | 0.11 | 0.01 | - | - |  |  |  |  |  |  |  |  |  |
| 35 | - | - | - | - | - | 0.08 | 0.44 | - | - | - | 0.40 | 0.13 | - | - | - | - | 0.25 | 0.51 | 0.30 |  |  |  |  |  |  |  |  |  |
| 36 | - | - | - | - | - | 0.07 | 0.18 | 0.15 | - | - | 0.47 | - | 0.17 | 0.23 | 0.43 | 0.14 | 0.22 | 0.31 | 0.30 |  |  |  |  |  |  |  |  |  |
| sum | 1 | 1 | 1 | 1 | 1 | 1 | 1 | 1 | 1 | 1 | 1 | 1 | 1 | 1 | 1 | 1 | 1 | 1 | 1 |  |  |  |  |  |  |  |  |  |
| **AMAR-AFM** | 1 | 2 | 3 | 4 | 7 | 11 | 13 | 14 | 15 | 17 | 21 | 22 | 23 | 25 | 26 | 27 | 28 | 29 | 30 | 31 | 32 | 33 | 34 |  |  |  |  |  |
| 1 | - | - | - | - | - | - | - | - | - | - | - | - | - | - | - | - | - | - | - | - | - | - | - |  |  |  |  |  |
| 2 | 0.10 | - | - | - | - | - | - | - | - | - | - | - | - | - | - | - | - | - | - | - | - | - | - |  |  |  |  |  |
| 3 | - | - | - | - | - | - | - | - | - | - | - | - | - | - | - | - | - | - | - | - | - | - | - |  |  |  |  |  |
| 4 | - | - | - | - | - | - | - | - | - | - | - | - | - | - | - | - | - | - | - | - | - | - | - |  |  |  |  |  |
| 7 | - | - | - | 0.00 | - | - | - | - | - | - | - | - | - | - | - | - | - | - | - | - | - | - | - |  |  |  |  |  |
| 11 | - | - | 0.08 | 0.00 | - | - | - | - | - | - | - | - | - | - | 0.04 | - | - | - | - | - | - | - | - |  |  |  |  |  |
| 13 | - | - | - | 0.00 | - | - | - | - | 0.01 | - | - | - | - | - | - | - | - | - | - | - | - | - | - |  |  |  |  |  |
| 14 | - | - | - | - | - | - | - | 0.02 | - | - | - | - | - | - | - | - | - | - | - | - | - | - | - |  |  |  |  |  |
| 15 | - | - | - | 0.00 | - | - | - | - | - | - | - | - | - | - | - | - | - | - | - | - | - | - | - |  |  |  |  |  |
| 17 | - | - | - | 0.00 | - | - | - | - | - | - | - | - | - | - | - | - | - | - | - | - | - | - | - |  |  |  |  |  |
| 21 | - | - | - | 0.00 | - | - | - | - | - | - | - | - | - | - | - | - | - | - | - | - | - | - | - |  |  |  |  |  |
| 22 | - | - | - | 0.01 | - | - | - | - | - | - | - | - | - | - | - | - | - | - | - | - | - | - | - |  |  |  |  |  |
| 23 | 0.10 | 0.26 | 0.09 | 0.01 | - | - | - | - | - | - | - | - | - | - | - | - | - | - | - | - | - | - | - |  |  |  |  |  |
| 25 | - | - | - | - | - | - | - | 0.18 | - | - | - | - | - | 0.06 | - | - | - | - | - | - | - | - | - |  |  |  |  |  |
| 26 | 0.38 | 0.01 | 0.04 | 0.01 | - | 0.02 | - | 0.06 | 0.40 | 0.02 | - | 0.21 | 0.26 | 0.03 | 0.14 | - | 0.07 | 0.00 | - | - | - | - | - |  |  |  |  |  |
| 27 | 0.29 | 0.02 | 0.12 | 0.01 | - | 0.02 | - | 0.16 | 0.17 | 0.14 | - | 0.21 | 0.15 | 0.04 | 0.26 | 0.00 | 0.06 | 0.00 | - | - | - | - | - |  |  |  |  |  |
| 28 | 0.08 | 0.05 | 0.12 | 0.01 | - | - | - | 0.01 | 0.07 | - | - | - | 0.01 | 0.11 | 0.01 | 0.01 | - | 0.01 | - | 0.02 | - | - | - |  |  |  |  |  |
| 29 | 0.01 | 0.17 | 0.05 | 0.01 | - | - | - | 0.10 | 0.08 | - | - | - | - | 0.02 | 0.15 | - | - | - | - | - | - | - | - |  |  |  |  |  |
| 30 | 0.01 | 0.22 | 0.05 | 0.01 | - | 0.30 | - | 0.20 | 0.10 | - | - | 0.20 | - | 0.03 | 0.08 | 0.08 | - | 0.00 | - | - | - | - | 0.05 |  |  |  |  |  |
| 31 | 0.01 | 0.24 | 0.27 | 0.01 | 0.11 | 0.08 | - | 0.19 | 0.05 | - | - | 0.18 | 0.49 | 0.02 | - | 0.09 | - | 0.01 | - | - | - | - | 0.05 |  |  |  |  |  |
| 32 | 0.02 | 0.03 | 0.06 | 0.67 | 0.02 | - | 0.25 | - | 0.12 | 0.17 | - | 0.00 | 0.00 | 0.02 | - | 0.01 | 0.24 | 0.03 | 0.29 | 0.22 | 0.05 | - | 0.25 |  |  |  |  |  |
| 33 | - | - | 0.12 | 0.25 | 0.20 | 0.44 | 0.25 | - | - | 0.31 | 0.43 | 0.14 | - | 0.53 | 0.15 | 0.81 | 0.25 | 0.68 | 0.06 | 0.53 | 0.47 | 0.18 | 0.05 |  |  |  |  |  |
| 34 | - | - | - | 0.01 | 0.67 | - | 0.35 | - | - | 0.25 | - | 0.06 | - | 0.02 | - | - | 0.35 | 0.03 | 0.22 | 0.11 | 0.01 | - | - |  |  |  |  |  |
| 35 | - | - | - | - | - | 0.08 | - | 0.08 | - | - | 0.40 | - | 0.08 | 0.13 | - | - | - | - | - | - | 0.25 | 0.51 | 0.30 |  |  |  |  |  |
| 36 | - | - | - | - | - | 0.07 | 0.15 | - | - | 0.12 | 0.17 | - | 0.01 | - | 0.17 | - | 0.05 | 0.23 | 0.43 | 0.12 | 0.22 | 0.31 | 0.30 |  |  |  |  |  |
| sum | 1 | 1 | 1 | 1 | 1 | 1 | 1 | 1 | 1 | 1 | 1 | 1 | 1 | 1 | 1 | 1 | 1 | 1 | 1 | 1 | 1 | 1 | 1 |  |  |  |  |  |
| **MAR-AFM** | 1 | 2 | 3 | 4 | 5 | 6 | 7 | 10 | 11 | 13 | 15 | 16 | 17 | 18 | 19 | 21 | 23 | 24 | 25 | 26 | 27 | 28 | 29 | 30 | 31 | 32 | 33 | 34 |
| 1 | - | - | - | - | - | - | - | - | - | - | - | - | - | - | - | - | - | - | - | - | - | - | - | - | - | - | - | - |
| 2 | 0.10 | - | - | - | - | - | - | - | - | - | - | - | - | - | - | - | - | - | - | - | - | - | - | - | - | - | - | - |
| 3 | - | - | - | - | - | - | - | - | - | - | - | - | - | - | - | - | - | - | - | - | - | - | - | - | - | - | - | - |
| 4 | - | - | - | - | - | - | - | - | - | - | - | - | - | - | - | - | - | - | - | - | - | - | - | - | - | - | - | - |
| 5 | - | - | - | 0.00 | 0.03 | - | - | - | - | - | - | - | - | - | - | - | - | - | - | - | - | - | - | - | - | - | - | - |
| 6 | - | - | - | 0.00 | - | - | - | - | - | - | - | - | - | - | - | - | - | - | - | - | - | - | - | - | - | - | - | - |
| 7 | - | - | - | 0.00 | - | - | - | - | - | - | - | - | - | - | - | - | - | - | - | - | - | - | - | - | - | - | - | - |
| 10 | - | - | - | - | - | - | - | 0.10 | - | - | - | - | - | - | - | - | - | - | - | - | - | - | - | - | - | - | - | - |
| 11 | - | - | 0.08 | 0.00 | 0.01 | - | - | 0.09 | - | - | - | - | - | - | - | - | - | - | - | 0.04 | - | - | - | - | - | - | - | - |
| 13 | - | - | - | 0.00 | 0.01 | - | - | 0.01 | - | - | 0.01 | - | - | - | - | - | - | - | - | - | - | - | - | - | - | - | - | - |
| 15 | - | - | - | 0.00 | - | - | - | - | - | - | - | - | - | - | - | - | - | - | - | - | - | - | - | - | - | - | - | - |
| 16 | - | - | - | 0.00 | - | - | - | - | - | - | - | 0.00 | - | - | - | - | - | - | - | - | - | - | - | - | - | - | - | - |
| 17 | - | - | - | - | - | - | - | - | - | - | - | - | - | - | - | - | - | - | - | - | - | - | - | - | - | - | - | - |
| 18 | - | - | 0.08 | 0.00 | - | - | - | - | - | - | - | 0.04 | - | - | - | - | - | - | - | - | - | - | - | - | - | - | - | - |
| 19 | - | - | - | 0.00 | - | - | - | - | - | - | - | - | 0.02 | - | - | - | - | - | - | - | - | - | - | - | - | - | - | - |
| 21 | - | - | - | - | - | - | - | - | - | - | - | - | - | - | 0.01 | - | - | - | - | - | - | - | - | - | - | - | - | - |
| 23 | 0.10 | 0.26 | 0.01 | 0.00 | 0.01 | 0.10 | - | 0.12 | - | - | - | 0.03 | - | - | - | - | - | - | - | - | - | - | - | - | - | - | - | - |
| 24 | - | - | - | - | - | 0.05 | - | 0.09 | - | - | - | 0.45 | - | - | - | - | - | - | - | - | - | - | - | - | - | - | - | - |
| 25 | - | - | - | - | - | - | - | 0.12 | - | - | - | - | - | - | - | - | - | - | 0.06 | - | - | - | - | - | - | - | - | - |
| 26 | 0.38 | 0.01 | 0.04 | 0.01 | 0.23 | 0.04 | - | 0.02 | 0.02 | - | 0.40 | 0.03 | - | 0.04 | - | - | 0.26 | 0.01 | 0.03 | 0.14 | - | 0.07 | 0.00 | - | - | - | - | - |
| 27 | 0.29 | 0.02 | 0.12 | 0.01 | 0.46 | 0.20 | - | 0.13 | 0.02 | - | 0.17 | 0.06 | 0.14 | 0.04 | 0.02 | - | 0.15 | 0.01 | 0.04 | 0.26 | 0.00 | 0.06 | 0.00 | - | - | - | - | - |
| 28 | 0.08 | 0.05 | 0.12 | 0.01 | 0.20 | 0.01 | - | 0.01 | - | - | - | 0.02 | - | - | - | - | 0.01 | - | 0.11 | 0.01 | 0.01 | - | 0.01 | - | 0.02 | - | - | - |
| 29 | 0.01 | 0.17 | 0.05 | 0.01 | - | 0.01 | - | 0.10 | - | - | - | - | - | - | - | - | - | - | 0.02 | 0.15 | - | - | - | - | - | - | - | - |
| 30 | 0.01 | 0.22 | 0.05 | 0.01 | - | 0.02 | - | 0.21 | 0.30 | - | 0.07 | 0.11 | - | - | - | - | - | - | 0.03 | 0.08 | 0.08 | - | 0.00 | - | - | - | - | 0.05 |
| 31 | 0.01 | 0.24 | 0.27 | 0.01 | - | 0.02 | 0.11 | - | 0.08 | - | 0.08 | 0.12 | - | - | 0.02 | - | 0.49 | 0.00 | 0.02 | - | 0.09 | - | 0.01 | - | - | - | - | 0.05 |
| 32 | 0.02 | 0.03 | 0.06 | 0.67 | - | 0.01 | 0.02 | - | - | 0.25 | 0.10 | 0.16 | 0.17 | 0.24 | 0.26 | - | 0.00 | 0.00 | 0.02 | - | 0.01 | 0.24 | 0.03 | 0.29 | 0.22 | 0.05 | - | 0.25 |
| 33 | - | - | 0.12 | 0.25 | - | 0.30 | 0.20 | 0.01 | 0.44 | 0.25 | 0.05 | - | 0.31 | 0.24 | 0.15 | 0.43 | - | 0.11 | 0.53 | 0.15 | 0.81 | 0.25 | 0.68 | 0.06 | 0.53 | 0.47 | 0.18 | 0.05 |
| 34 | - | - | - | 0.01 | - | 0.25 | 0.67 | - | - | 0.35 | 0.12 | - | 0.25 | 0.46 | 0.18 | - | - | - | 0.02 | - | - | 0.35 | 0.03 | 0.22 | 0.11 | 0.01 | - | - |
| 35 | - | - | - | - | 0.01 | - | - | - | 0.08 | - | - | - | - | - | 0.10 | 0.40 | 0.08 | 0.40 | 0.13 | - | - | - | - | - | - | 0.25 | 0.51 | 0.30 |
| 36 | - | - | - | - | 0.05 | - | - | - | 0.07 | 0.15 | - | - | 0.12 | - | 0.26 | 0.17 | 0.01 | 0.47 | - | 0.17 | - | 0.05 | 0.23 | 0.43 | 0.12 | 0.22 | 0.31 | 0.30 |
| sum | 1 | 1 | 1 | 1 | 1 | 1 | 1 | 1 | 1 | 1 | 1 | 1 | 1 | 1 | 1 | 1 | 1 | 1 | 1 | 1 | 1 | 1 | 1 | 1 | 1 | 1 | 1 | 1 |
| **FAFAR-AFM** | 1 | 2 | 3 | 4 | 7 | 8 | 11 | 13 | 15 | 17 | 18 | 20 | 23 | 24 | 25 | 26 | 27 | 28 | 29 | 30 | 31 | 32 | 33 | 34 |  |  |  |  |
| 1 | - | - | - | - | - | - | - | - | - | - | - | - | - | - | - | - | - | - | - | - | - | - | - | - |  |  |  |  |
| 2 | 0.10 | - | - | - | - | - | - | - | - | - | - | - | - | - | - | - | - | - | - | - | - | - | - | - |  |  |  |  |
| 3 | - | - | - | - | - | - | - | - | - | - | - | - | - | - | - | - | - | - | - | - | - | - | - | - |  |  |  |  |
| 4 | - | - | - | - | - | - | - | - | - | - | - | - | - | - | - | - | - | - | - | - | - | - | - | - |  |  |  |  |
| 7 | - | - | - | 0.00 | - | - | - | - | - | - | - | - | - | - | - | - | - | - | - | - | - | - | - | - |  |  |  |  |
| 8 | - | - | - | 0.00 | - | - | - | - | - | - | - | - | - | - | - | - | - | - | - | - | - | - | - | - |  |  |  |  |
| 11 | - | - | 0.08 | 0.01 | - | - | - | - | - | - | - | - | - | - | - | 0.04 | - | - | - | - | - | - | - | - |  |  |  |  |
| 13 | - | - | - | 0.01 | - | - | - | - | 0.01 | - | - | - | - | - | - | - | - | - | - | - | - | - | - | - |  |  |  |  |
| 15 | - | - | - | 0.00 | - | - | - | - | - | - | - | - | - | - | - | - | - | - | - | - | - | - | - | - |  |  |  |  |
| 17 | - | - | - | - | - | - | - | - | - | - | - | - | - | - | - | - | - | - | - | - | - | - | - | - |  |  |  |  |
| 18 | - | - | 0.08 | 0.01 | - | - | - | - | - | - | - | - | - | - | - | - | - | - | - | - | - | - | - | - |  |  |  |  |
| 20 | - | - | - | 0.00 | - | - | - | - | - | - | - | - | - | - | - | - | - | - | - | - | - | - | - | - |  |  |  |  |
| 23 | 0.10 | 0.26 | 0.01 | 0.00 | - | - | - | - | - | - | - | - | - | - | - | - | - | - | - | - | - | - | - | - |  |  |  |  |
| 24 | - | - | - | - | - | - | - | - | - | - | - | - | - | - | - | - | - | - | - | - | - | - | - | - |  |  |  |  |
| 25 | - | - | - | - | - | - | - | - | - | - | - | - | - | - | 0.06 | - | - | - | - | - | - | - | - | - |  |  |  |  |
| 26 | 0.38 | 0.01 | 0.04 | 0.01 | - | - | 0.02 | - | 0.40 | 0.02 | 0.04 | - | 0.26 | 0.01 | 0.03 | 0.14 | - | 0.07 | 0.00 | - | - | - | - | - |  |  |  |  |
| 27 | 0.29 | 0.02 | 0.12 | 0.01 | - | - | 0.02 | - | 0.17 | 0.14 | 0.04 | - | 0.15 | 0.01 | 0.04 | 0.26 | 0.00 | 0.06 | 0.00 | - | - | - | - | - |  |  |  |  |
| 28 | 0.08 | 0.05 | 0.12 | 0.01 | - | - | - | - | - | - | - | - | 0.01 | - | 0.11 | 0.01 | 0.01 | - | 0.01 | - | 0.02 | - | - | - |  |  |  |  |
| 29 | 0.01 | 0.17 | 0.05 | 0.01 | - | - | - | - | - | - | - | - | - | - | 0.02 | 0.15 | - | - | - | - | - | - | - | - |  |  |  |  |
| 30 | 0.01 | 0.22 | 0.05 | 0.01 | - | - | 0.30 | - | 0.07 | - | - | - | - | - | 0.03 | 0.08 | 0.08 | - | 0.00 | - | - | - | - | 0.05 |  |  |  |  |
| 31 | 0.01 | 0.24 | 0.27 | 0.01 | 0.11 | - | 0.08 | - | 0.08 | - | - | - | 0.49 | 0.00 | 0.02 | - | 0.09 | - | 0.01 | - | - | - | - | 0.05 |  |  |  |  |
| 32 | 0.02 | 0.03 | 0.06 | 0.67 | 0.02 | - | - | 0.25 | 0.10 | 0.17 | 0.24 | - | 0.00 | 0.00 | 0.02 | - | 0.01 | 0.24 | 0.03 | 0.29 | 0.22 | 0.05 | - | 0.25 |  |  |  |  |
| 33 | - | - | 0.12 | 0.25 | 0.20 | 0.20 | 0.44 | 0.25 | 0.05 | 0.31 | 0.24 | 0.85 | - | 0.11 | 0.53 | 0.15 | 0.81 | 0.25 | 0.68 | 0.06 | 0.53 | 0.47 | 0.18 | 0.05 |  |  |  |  |
| 34 | - | - | - | 0.01 | 0.67 | - | - | 0.35 | 0.12 | 0.25 | 0.46 | - | - | - | 0.02 | - | - | 0.35 | 0.03 | 0.22 | 0.11 | 0.01 | - | - |  |  |  |  |
| 35 | - | - | - | - | - | 0.80 | 0.08 | - | - | - | - | 0.15 | 0.08 | 0.40 | 0.13 | - | - | - | - | - | - | 0.25 | 0.51 | 0.30 |  |  |  |  |
| 36 | - | - | - | - | - | - | 0.07 | 0.15 | - | 0.12 | - | - | 0.01 | 0.47 | - | 0.17 | - | 0.05 | 0.23 | 0.43 | 0.12 | 0.22 | 0.31 | 0.30 |  |  |  |  |
| sum | 1 | 1 | 1 | 1 | 1 | 1 | 1 | 1 | 1 | 1 | 1 | 1 | 1 | 1 | 1 | 1 | 1 | 1 | 1 | 1 | 1 | 1 | 1 | 1 |  |  |  |  |

**Table S5 Comparison of trophic level parameters of food web functional groups of seven different** **spatio-temporal models in PRE marine pasture in 2020.**

| Group name | AWAR-  BFM | WAR-  BFM | AWAR-  AFM | WAR-  AFM | AMAR-  AFM | MAR-  AFM | FAFAR-  AFM |
| --- | --- | --- | --- | --- | --- | --- | --- |
| 1.Marine mammals | 4.69 | 4.71 | 4.71 | 4.29 | 4.71 | 4.71 | 4.71 |
| 2.Chondrichthyes | 4.36 | 4.39 | 4.39 | 4.03 | 4.39 | 4.39 | 4.39 |
| 3.Fish-eating birds | 4.20 | 4.20 | 4.20 | 4.01 | 4.24 | 4.20 | 4.20 |
| 4.Flatfishes | 3.61 | 3.61 | 3.61 | 3.60 | 3.62 | 3.62 | 3.61 |
| 5.Saurida tumbil | - | 4.42 | - | - | - | 4.42 | - |
| 6.Trachurus japonicus | - | - | - | - | - | 3.88 | - |
| 7.Argyrosomus argentatus | 3.66 | 3.66 | 3.66 | 3.65 | 3.66 | 3.66 | 3.66 |
| 8.Psenopsis anomala | - | - | - | - | - | - | 2.24 |
| 9.Kammal thryssa | - | 3.21 | - | - | - | - | - |
| 10.Anglerfish | - | - | - | - | - | 4.34 | - |
| 11.Other gobiidae | 3.38 | 3.38 | 3.38 | 3.37 | 3.38 | 3.38 | 3.38 |
| 12.Secutor ruconius | - | - | - | 2.59 | - | - | - |
| 13.Other Cynoglossidae | 3.31 | 3.31 | 3.31 | 3.31 | 3.31 | 3.31 | 3.31 |
| 14.Other Trichiurus | - | - | 3.97 | - | 4.09 | - | - |
| 15.Scorpaenidae | 4.04 | 4.28 | 4.28 | 4.22 | 4.38 | 4.28 | 4.28 |
| 16.Other Synodidae | - | - | 3.72 | - | - | 3.72 | - |
| 17.Tetraodontidae | - | - | - | - | 3.46 | 3.45 | 3.46 |
| 18.Other Sciaenidae | 3.64 | 3.64 | 3.64 | 3.63 | - | 3.64 | 3.64 |
| 19.Mugilidae | - | - | - | - | - | 3.03 | - |
| 20.Clupeidae | - | - | - | - | - | - | 3.04 |
| 21.Engraulidae | - | - | - | - | 2.53 | 2.53 | - |
| 22.Anguilliformes | 4.15 | - | - | - | 4.15 | - | - |
| 23.Other Piscivorous fishes | 4.07 | 4.21 | 4.21 | - | 4.21 | 4.21 | 4.21 |
| 24.Other omnivorous fishes | 2.19 | - | 2.19 | 2.18 | - | 2.19 | 2.19 |
| 25.Other demersal fish | - | - | 3.44 | 3.23 | 3.44 | 3.44 | 3.44 |
| 26.Other benthic fishes 1 | 3.77 | 3.77 | 3.77 | 3.71 | 3.77 | 3.77 | 3.77 |
| 27.Other benthic fishes 2 | 3.39 | 3.39 | 3.39 | - | 3.39 | 3.39 | 3.39 |
| 28.Cephalopods | 3.60 | 3.60 | 3.60 | - | 3.60 | 3.60 | 3.60 |
| 29.Mantis shrimp | 2.99 | 2.99 | 2.99 | 2.99 | 2.99 | 2.99 | 2.99 |
| 30.Shrimps | 2.93 | 2.93 | 2.93 | 2.93 | 2.93 | 2.93 | 2.93 |
| 31.Crabs | 3.25 | 3.25 | 3.25 | 3.20 | 3.25 | 3.25 | 3.25 |
| 32.Mollusks | 2.67 | 2.67 | 2.67 | 2.67 | 2.67 | 2.67 | 2.67 |
| 33.Other Zooplankton | 2.22 | 2.22 | 2.22 | 2.22 | 2.22 | 2.22 | 2.22 |
| 34.Gastropoda | 2.69 | 2.69 | 2.69 | 2.69 | 2.69 | 2.69 | 2.69 |
| 35.Phytoplankton | 1.00 | 1.00 | 1.00 | 1.00 | 1.00 | 1.00 | 1.00 |
| 36.Detritus | 1.00 | 1.00 | 1.00 | 1.00 | 1.00 | 1.00 | 1.00 |

**Reference:**

Chen, Z. Z., Xu, S. N., and Qiu, Y. S. 2015. Using a food-web model to assess the trophic structure and energy flows in Daya Bay, China. Continental Shelf Research, 111: 316-326.

Duan, L. J., Li, S. Y., Liu, Y., Jiang, T., and Failler, P. 2009a. A trophic model of the Pearl River Delta coastal ecosystem. Ocean & Coastal Management, 52: 359-367.

Duan, L. J., Li, S. Y., Liu, Y., Moreau, J., and Christensen, V. 2009b. Modeling changes in the coastal ecosystem of the Pearl River Estuary from 1981 to 1998. Ecological Modelling, 220: 2802-2818.

Lee, S. I., and Zhang, C. I. 2018. Evaluation of the Effect of Marine Ranching Activities on the Tongyeong Marine Ecosystem. Ocean Science Journal, 53: 557-582.

Rahman, M. F., Qun, L., Shan, X., Chen, Y., Ding, X., and Liu, Q. 2019. Temporal Changes of Structure and Functioning of the Bohai Sea Ecosystem: Insights from Ecopath Models. Thalassas, 35: 625-641.

Sun, L. Q., Lin, Y. S., Chen, L. X., Cao, W. Q., and Zheng, L. M. 2016. Analysis of ecosystem structure and function in the northern Beibu Gulf Ⅶ: Nutrition structure and keystone species selection based on Ecopath with Ecosim (Translation, in Chinese). Journal of Tropical Oceanography, 35: 51-62.
